# A human STAT3 gain-of-function variant confers T cell dysregulation without predominant Treg dysfunction in mice

**DOI:** 10.1172/jci.insight.162695

**Published:** 2022-11-08

**Authors:** Erica G. Schmitt, Kelsey A. Toth, Samuel I. Risma, Ana Kolicheski, Nermina Saucier, Rafael J. Feliciano Berríos, Zev J. Greenberg, Jennifer W. Leiding, Jack J. Bleesing, Akaluck Thatayatikom, Laura G. Schuettpelz, John R. Edwards, Tiphanie P. Vogel, Megan A. Cooper

**Affiliations:** 1 Department of Pediatrics, Division of Rheumatology and Immunology,; 2 Center for Pharmacogenomics, Department of Medicine, and; 3 Department of Pediatrics, Division of Hematology and Oncology, Washington University School of Medicine, St. Louis, Missouri, USA.; 4 Division of Allergy and Immunology, Department of Pediatrics, Johns Hopkins University, Baltimore, Maryland.; 5 Infectious Diseases and Immunology, Arnold Palmer Hospital for Children, Orlando, Florida, USA.; 6 Division of BM Transplantation and Immune Deficiency, Cincinnati Children’s Hospital Medical Center, Department of Pediatrics, University of Cincinnati College of Medicine, Cincinnati, Ohio, USA.; 7 AdventHealth for Children, Orlando, Florida, USA.; 8 Division of Rheumatology, Department of Pediatrics, Baylor College of Medicine and Texas Children’s Hospital, Houston, Texas, USA.; 9 Department of Pathology and Immunology, Washington University School of Medicine, St. Louis, Missouri, USA.

**Keywords:** Immunology, Autoimmune diseases, Genetic diseases, T cells

## Abstract

Primary immune regulatory disorders (PIRD) represent a group of disorders characterized by immune dysregulation, presenting with a wide range of clinical disease, including autoimmunity, autoinflammation, or lymphoproliferation. Autosomal dominant germline gain-of-function (GOF) variants in *STAT3* result in a PIRD with a broad clinical spectrum. Studies in patients have documented a decreased frequency of FOXP3^+^ Tregs and an increased frequency of Th17 cells in some patients with active disease. However, the mechanisms of disease pathogenesis in STAT3 GOF syndrome remain largely unknown, and treatment is challenging. We developed a knock-in mouse model harboring a de novo pathogenic human STAT3 variant (p.G421R) and found these mice developed T cell dysregulation, lymphoproliferation, and CD4^+^ Th1 cell skewing. Surprisingly, Treg numbers, phenotype, and function remained largely intact; however, mice had a selective deficiency in the generation of iTregs. In parallel, we performed single-cell RNA-Seq on T cells from STAT3 GOF patients. We demonstrate only minor changes in the Treg transcriptional signature and an expanded, effector CD8^+^ T cell population. Together, these findings suggest that Tregs are not the primary driver of disease and highlight the importance of preclinical models in the study of disease mechanisms in rare PIRD.

## Introduction

Immune dysregulation arises when there is a disruption of immunological tolerance or alterations in the mechanisms utilized to downregulate the immune response after an insult. Discovery of monogenic inborn errors of immunity (IEI), presenting predominantly with immune dysregulation rather than susceptibility to infection, have led to important findings about key factors regulating the human immune response. These disorders, known as primary immune regulatory disorders (PIRD), present with a wide range of clinical disease — for example, early-onset autoimmune disease, hemophagocytic lymphohistiocytosis (HLH), autoinflammatory syndromes, very early–onset inflammatory bowel disease, and/or lymphoproliferation (1–3). Loss of T cell tolerance is a mechanism for several PIRD. For instance, disruption of Tregs due to deleterious variants in the *FOXP3* gene result in immune dysregulation, polyendocrinopathy, enteropathy, X-linked syndrome (IPEX syndrome), which is characterized by multiorgan autoimmunity due to loss of peripheral tolerance from absent or dysfunctional Tregs (4–9). IPEX syndrome was the first PIRD characterized in a group of monogenic disorders resulting in Treg defects, sometimes termed “Tregopathies” (10). Discovery and investigation of pathogenic human *FOXP3* variants has provided key insights into structural protein domains and the function of FOXP3. As another example, patients with autoimmune polyendocrinopathy with candidiasis and ectodermal dystrophy (APECED) were found to have defects in the gene *AIRE*, encoding an important transcription factor, a discovery that ultimately led to insights into thymic expression of tissue-specific transcripts and central tolerance (11–13). Interestingly, while there is clinical overlap between IPEX and APECED — for example, endocrinopathies — the means by which T cell tolerance is altered are quite distinct, demonstrating how monogenic disease with clinical overlap can have different immunologic mechanisms. Thus, studying PIRD is not only instrumental for elucidating disease pathogenesis and treatment strategies for individual patients, but it also can provide context for understanding key immunological mechanisms.

Autosomal dominant germline gain-of-function (GOF) variants in STAT3 result in a PIRD characterized by immune dysregulation and a broad spectrum of clinical features, including cytopenias, lymphadenopathy, interstitial lung disease, enteropathy, and polyendocrinopathy (14, 15). STAT3 is a highly conserved transcription factor downstream of multiple cytokines and growth factors, and signaling through activated, phosphorylated STAT3 (p-STAT3) homodimers is implicated in both pro- and antiinflammatory pathways (16). STAT3 is also a key transcription factor involved in the regulation and balance of the Treg/Th17 cell polarization axis (17). A decreased frequency of FOXP3^+^ Tregs has been observed in the peripheral blood of some, but not all, STAT3 GOF patients, and taken together with the overlapping clinical features in STAT3 GOF and IPEX syndrome, it has been suggested that this disease falls within the spectrum of Tregopathies (10, 14). However, whether decreased numbers of Tregs in the peripheral blood are a primary cause of disease and if there are intrinsic defects in Treg generation, as in IPEX syndrome, is unknown. Confirming Treg defects as a primary cause of disease pathogenesis would have an impact on the approach to treatment in patients with STAT3 GOF syndrome — for instance, cell-based therapies, such as autologous gene therapy, or Treg-based therapies.

To further investigate the role of STAT3 GOF in immune dysregulation and Tregs, we developed a mouse model of STAT3 GOF with a DNA-binding domain variant identified in patients, p.G421R, on the C57BL/6 background. Patients with this variant have presented with multiorgan involvement, including autoimmune cytopenias, lymphadenopathy, hepatosplenomegaly, autoimmune hepatitis, scleroderma, polyarthritis, respiratory infections, enteropathy, and short stature (14, 18–20). Using this mouse model, we performed a series of phenotypic and functional studies, focusing on Tregs, given the previously noted Treg deficiency in patients. We found that the variant imparts progressive lymphoproliferation in affected mice, with an expansion of CD4 and CD8 T effector memory (TEM) cells. Interestingly, STAT3 GOF mice had normal to increased Treg numbers, but the ability to generate induced Tregs (iTregs) was selectively impaired. STAT3 GOF Tregs were otherwise similar to WT, with only minor differences seen in disease models and transcriptional signatures. Surprisingly, further analysis of CD4 subsets in a colitis disease model and BM chimera demonstrated a Th1-skewed effector CD4 T cell compartment, but there was no increase in Th17 cells. Finally, we examined the transcriptional signature of humans with STAT3 GOF syndrome using single-cell RNA-Seq. Collectively, these data have implications for disease paradigms and treatment targets going forward — specifically, Treg-centered therapies may be insufficient in STAT3 GOF syndrome, and there appears to be a Th1 signature in this disease model.

## Results

### Heterozygous G421R mice recapitulate aspects of human disease.

**Figure 1:**
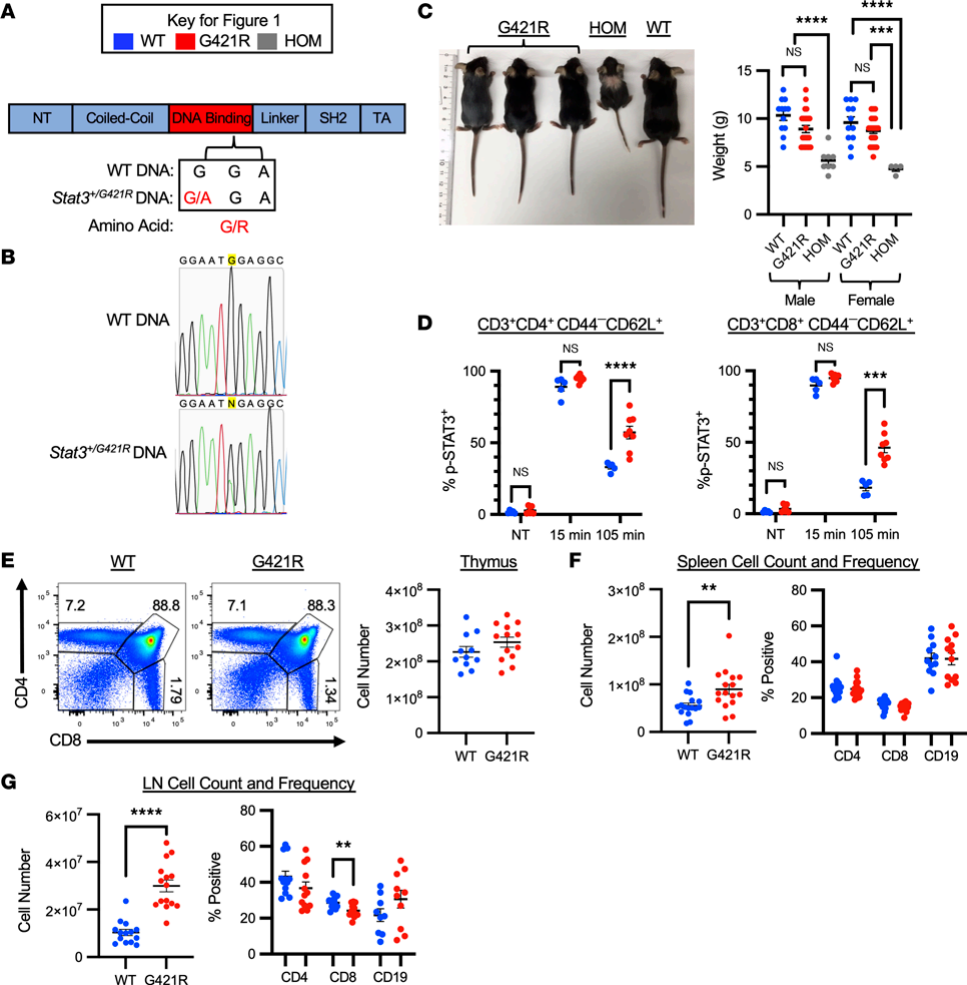
Generation of the STAT3 GOF mice. (**A**) STAT3 p.G421R variant located in the DNA-binding domain was inserted into WT mice using CRISPR-Cas9. NT, N-terminal; TA, transactivation domain. (**B**) The point mutation was confirmed by Sanger sequencing. (**C**) WT, G421R (*Stat3*^p.G421R/+^) and HOM (*Stat3*^p.G421R/^ ^p.G421R^) littermates at 38 days of age (left) and weaning weight of male and female littermates (right). (**D**) Splenocytes from WT and G421R mice were stimulated with IL-6 for 15 minutes, washed, and then analyzed or returned to culture for the indicated time prior to analysis for p-STAT3. (**E**) Representative flow cytometry from the thymus of 3- to 4-week-old mice (left) and thymus cell counts (right). (**F**) Scatter plot showing adult mice spleen cell counts and frequency of CD3^+^CD4^+^, CD3^+^CD8^+^, and CD3^–^CD19^+^ cells within the live cell gate. (**G**) Scatter plot showing adult mice pooled peripheral lymph node (axillary, brachial, inguinal) cell counts and frequency of CD3^+^CD4^+^, CD3^+^CD8^+^, and CD3^–^CD19^+^ cells within the live cell gate. For all scatter plots, each dot represents an individual mouse, and data are shown as mean ± SEM. Data are representative of at least 3 independent experiments, except for **D**, which represents 2 independent experiments. Young mice, <6 weeks of age; adult mice, 7–16 weeks of age; old mice, > 20 weeks of age. An unpaired *t* test was used for all comparisons with 2 groups, and Welch’s *t* test was used in the instance of unequal variance; for those with 3 or more groups, 1-way ANOVA was used, except for in **D**, which was analyzed with a 2-way ANOVA. **P < 0.01, ***P < 0.001, ****P < 0.0001.

To investigate the pathogenesis of STAT3 GOF syndrome, we developed a mouse model of the disease-causing p.G421R DNA-binding domain variant. Mice were generated using CRISPR/CAS9 technology to introduce a single nucleotide change (Figure 1A). The point mutation, c.1261G>A, was verified by Sanger sequencing (Figure 1B), and mice were fully back-crossed to the C57BL/6 background. Male and female mice heterozygous for the p.G421R variant (*Stat3*^p.G421R/+^, subsequently referred to as STAT3 GOF or G421R mice) had a similar weaning weight compared with WT counterparts, and survival and breeding were not impaired (Figure 1C). Interestingly, mice homozygous for the p.G421R variant were runted and died shortly after the time of weaning (Figure 1C). To determine the impact of the p.G421R variant on STAT3 activity in heterozygous mice, STAT3 phosphorylation was measured in naive splenic CD4^+^ and CD8^+^ T cells. At baseline or with IL-6 stimulation, p-STAT3 was similar in splenic T cells from STAT3 GOF mice and littermate controls (Figure 1D). However, consistent with findings in a patient with the p.G421R variant, there was delayed dephosphorylation of STAT3 in T cells from STAT3 GOF mice (Figure 1D) (14). Collectively, these data demonstrate that the p.G421R variant results in STAT3 GOF in mice.

The immune phenotype of the mice was analyzed at different stages of development, including young (<6 weeks of age), adult (7–16 weeks of age), and old (>20 weeks of age) mice. Young mice had normal counts and populations of CD4 and CD8 T cells develop in the thymus (Figure 1E and Supplemental Figure 1A; supplemental material available online with this article; https://doi.org/10.1172/jci.insight.162695DS1). Thymic CD4 and CD8 T cell frequencies and CD4 SP and CD8 single positive (SP) T cell maturation were similar, on a global level, in WT and STAT3 GOF mice (Supplemental Figure 1B). Unexpectedly, mice homozygous for the STAT3 p.G421R variant had a significantly reduced thymus size (Supplemental Figure 1C).

Young STAT3 GOF mice (<6 weeks of age) had a normal spleen size (Supplemental Figure 1D); however, by adulthood (age 7–16 weeks), significant splenomegaly was observed (Figure 1F). A similar frequency of CD4, CD8, and CD19 lymphocytes were observed in the spleens of WT and STAT3 GOF mice, but there were increased numbers of these cell subsets (Figure 1F; data not shown). Aged mice (>20 weeks) demonstrated a similar pattern of splenomegaly and increased numbers of T and B lymphocytes (data not shown). STAT3 GOF mice also developed lymphadenopathy as they reached maturity (Figure 1G), with a reduction in the CD8 T cell frequency but overall increased numbers of CD4, CD8, and CD19 lymphocytes (data not shown). In the lymph nodes (LNs) of aged mice, there was a relative decrease in the frequency of T cells (CD4 and CD8) and an increase in the frequency of CD19^+^ B cells; however, cell counts for all of these populations were increased in the STAT3 GOF mice (Supplemental Figure 1E; data not shown).

**Figure 2:**
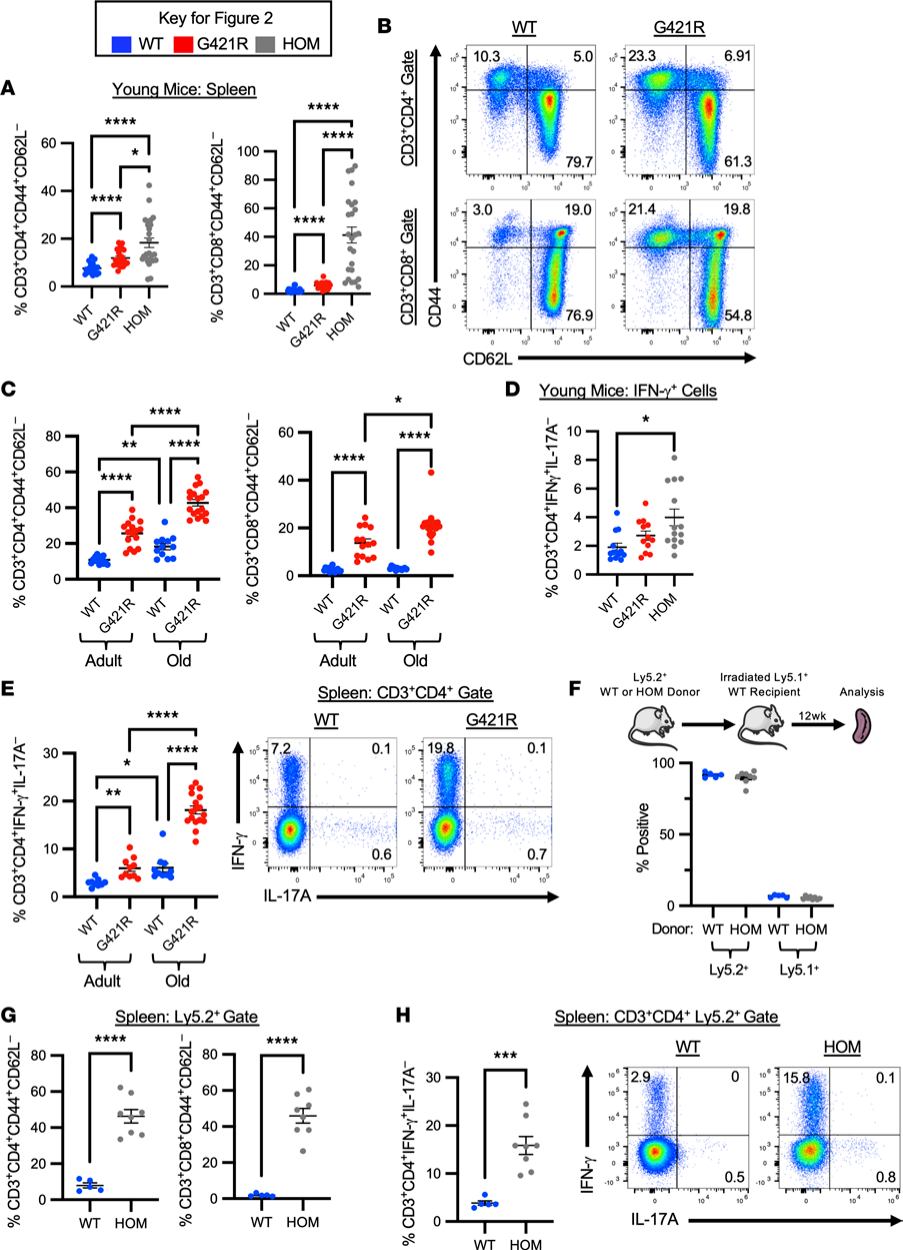
T cell dysregulation in STAT3 GOF mice. (**A**) Frequency of CD3^+^CD4^+^CD44^+^CD62L^–^ or CD3^+^CD8^+^CD44^+^CD62L^–^ T cells in the spleen of young mice. (**B**) Representative flow cytometry from the spleen of adult mice. (**C**) Frequency of CD3^+^CD4^+^CD44^+^CD62L^–^ or CD3^+^CD8^+^CD44^+^CD62L^–^ T cells in the spleen of adult and old mice. (**D**) Frequency of CD3^+^CD4^+^IFN-γ^+^IL-17A^–^ cells in the spleen of young mice. (**E**) Scatter plot showing adult and old mice spleen frequency of CD3^+^CD4^+^IFN-γ^+^IL-17A^–^ cells (left), and representative flow cytometry from the spleen of old mice (right). (**F**) Graphical representation of experimental outline for BM transplant (top). Frequency of Ly5.2^+^ or Ly5.1^+^ cells in the spleen of transplanted mice, gated on live, single cells. Data are representative of 3–4 independent experiments; *n* = 8 (HOM → WT) and *n* = 5 (WT → WT). (**G**) Frequency of donor Ly5.2^+^ cells that were CD3^+^CD4^+^CD44^+^CD62L^–^ or CD3^+^CD8^+^CD44^+^CD62L^–^ in the spleen of transplanted mice. (**H**) Percentage of donor Ly5.2^+^ cells that were CD3^+^CD4^+^IFN-γ^+^IL-17A^–^ (left) and representative flow cytometry from the spleen of transplanted mice (right). For all scatter plots, each dot represents an individual mouse, and data are shown as mean ± SEM. Data are representative of at least 3 independent experiments. An unpaired *t* test was used for all comparisons with 2 groups, and Welch’s *t* test was used in the instance of unequal variance; for those with 3 or more groups, 1-way ANOVA was used. *P < 0.05, **P < 0.01, ***P < 0.001, ****P < 0.0001.

While young STAT3 GOF mice did not have lymphoproliferation, there was evidence of immune dysregulation at this early time point, with an increased frequency of CD44^+^CD62L^–^ activated effector CD4 and CD8 T cells (Figure 2A). These cells continued to accumulate and increased with aging (Figure 2, B and C). STAT3 GOF mice had a significant increase in CD3^+^CD4^+^CD44^+^ cells that were Ki-67^+^, suggesting increased proliferation of this population (Supplemental Figure 1F). Splenic T cells from mice homozygous for the STAT3 GOF variant also displayed an activated, effector phenotype at a young age (Figure 2A). Overall, these data suggest that STAT3 GOF mice recapitulate many aspects of STAT3 GOF syndrome, including lymphoproliferation with splenomegaly and lymphadenopathy.

CD4^+^ T cells isolated from patients with STAT3 GOF syndrome have been shown to produce proinflammatory cytokines, such as IL-17, and this pathway has garnered particular interest, given the role of STAT3 in Th17 cell development (14). In at least 1 instance, however, CD4^+^ T cells from patients with STAT3 GOF syndrome have also been shown to produce IFN-γ with stimulation (21). Therefore, we examined cytokine production in STAT3 GOF mice in response to ex vivo stimulation with PMA/Ionomycin. Unexpectedly, there was an increased frequency of IFN-γ–producing cells among splenic CD4^+^ T cells isolated from mice homozygous for the STAT3 GOF variant (Figure 2D). In vitro differentiation of naive T cells under Th1-polarizing conditions revealed a mild reduction in the ability of STAT3 GOF T cells to differentiate into IFN-γ–producing cells (Supplemental Figure 1G). In ex vivo–stimulated cells, CD4^+^ T cell Th1 polarization was seen in adult mice heterozygous for the variant, and it was further exaggerated in aged mice (Figure 2E). There were no differences in ex vivo IL-17A–producing cells. These data reveal a dysregulated, Th1 response in STAT3 GOF mice.

To study whether the observed T cell phenotype was cell intrinsic, BM chimeras were generated. Congenic Ly5.1^+^ lethally irradiated hosts were reconstituted with BM from WT or homozygous STAT3 GOF mice (Ly5.2^+^). Mice were analyzed 12 weeks after reconstitution. Mice transplanted with homozygous BM survived to the conclusion of the experiment and had good engraftment of donor BM in the peripheral blood by 10 weeks after transplant (Supplemental Figure 1H). Similar to mice receiving WT BM, splenocytes isolated from mice transplanted with BM from homozygous mice were comprised largely of donor Ly5.2^+^ cells (Figure 2F). Strikingly, the dysregulated T cell phenotype was preserved. Mice that received homozygous STAT3 GOF BM had an increased frequency of CD44^+^CD62L^–^ activated effector CD4 and CD8 T cells in the spleen (Figure 2G). There was also an increased frequency of IFN-γ–producing cells among splenic CD4^+^ T cells derived from STAT3 GOF homozygous donors (Figure 2H). This suggests that T cell–intrinsic defects contribute to the observed phenotype in STAT3 GOF mice.

### STAT3 GOF Tregs are not substantially altered.

**Figure 3:**
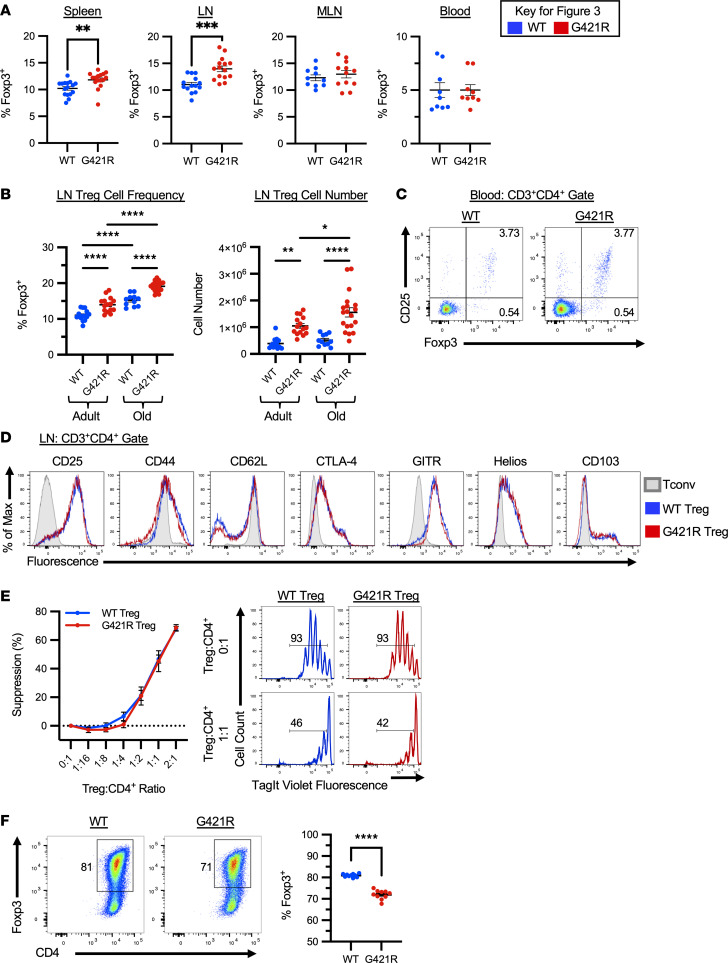
Tregs in STAT3 GOF mice. (**A**) Frequency of CD3^+^CD4^+^Foxp3^+^ Tregs in the indicated tissues of adult mice. (**B**) Percentage (left) and number (right) of CD3^+^CD4^+^Foxp3^+^ Tregs in the peripheral lymph node of adult and old mice. (**C**) Representative flow cytometry from the peripheral blood of adult mice. (**D**) Representative flow cytometry from the peripheral lymph node showing staining for the indicated markers, gated on WT conventional T cells (T_conv_) (CD3^+^CD4^+^EGFP^–^), or Tregs (CD3^+^CD4^+^EGFP^+^) from WT and G421R mice. Data are representative of at least 9 mice and 4 independent experiments. (**E**) Suppressive capacity of WT and G421R Tregs as measured by Tag-it Violet dilution on responder T cells. Graph depicting the mean ± SEM of the percent suppression, at each Treg/CD4 ratio (left). Representative histograms are shown along with the average proliferation of CD4^+^ T cells for selected Treg to CD4 ratios (right). Percent suppression = (percent proliferation of the CD4^+^ T cells at the 0:1 Treg/Teff ratio — percent proliferation of CD4^+^ T cells for each Treg to CD4 ratio)/percent proliferation of the CD4^+^ T cells at the 0:1 Treg/Teff ratio. Data were from 3 experiments consisting of 8–9 individual assays. (**F**) In vitro–derived iTregs were generated from WT or G421R CD4^+^EGFP^–^CD45RB^hi^ naive T cells with anti-CD3, anti-CD28, TGF-β1, and IL-2. Representative flow cytometry (left) and scatter plot showing the frequency of CD4^+^Foxp3^+^ Tregs (right). Each point represents an individual mouse and at least 3 independent experiments. An unpaired *t* test was used for comparisons with 2 groups, a Welch’s *t* test was used for unequal variance, and — for 3 or more groups — 1-way ANOVA was used, except for in **E**, which was analyzed with 2-way ANOVA. *P < 0.05, **P < 0.01, ***P < 0.001, ****P < 0.0001.

Prior studies in humans with STAT3 GOF syndrome suggest that these patients may have defects in FOXP3^+^ Tregs; this is based on studies demonstrating reduced Treg frequency in the peripheral blood of patients, organ-specific autoimmunity (i.e., enteropathy, type 1 diabetes, cytopenias), and in some instances, reduced Treg suppressive capacity in vitro (10, 14, 15, 21, 22). To characterize Tregs in STAT3 GOF mice, mice were crossed to *Foxp3*^EGFP^ reporter mice, which accurately identifies Foxp3^+^ Tregs with EGFP (23). The frequency and number of Tregs was similar in the thymus of WT and STAT3 GOF mice (Supplemental Figure 2A). Interestingly, an increased frequency of Tregs was seen in the thymus of STAT3 GOF homozygous mice, though given the reduction in the size of the thymus, Treg numbers were significantly reduced (Supplemental Figure 2A). Treg frequency and number were similar in the spleen of young mice (Supplemental Figure 2B). In adult mice, Treg frequency and numbers were normal — and, in some cases, increased — in the tissues examined: spleen, peripheral LN, mesenteric LN (MLN), and blood (Figure 3A). With aging, there was a progressive increase in the frequency and number of Tregs in the peripheral LNs (Figure 3B). Aged STAT3 GOF mice also accumulated an increased number of Tregs in the spleen (Supplemental Figure 2C). In contrast to some STAT3 GOF patients with active disease, peripheral blood Foxp3^+^CD25^+^ Treg frequencies were normal in this model (Figure 3C).

The phenotype of the STAT3 GOF Tregs was assayed by flow cytometric analysis of several canonical Treg markers. The frequency of Tregs in the LN expressing each marker, as well as the median fluorescence intensity of CD25, CD44, CD62L, CTLA-4, Helios, and CD103, were similar between WT and STAT3 GOF Tregs (Figure 3D). However, there was a small but significantly increased frequency of GITR^+^ Tregs in the LN of STAT3 GOF mice, and the MFI of GITR was also increased on these Tregs (Supplemental Figure 2D). There was also an increased frequency of CD103^+^ Tregs in the spleen of STAT3 GOF mice, and the MFI level of CD103 was similarly elevated (Supplemental Figure 2E). The function of ex vivo–isolated STAT3 GOF Tregs was assessed using an in vitro suppression assay, with suppressive capability measured by the ability of Tregs to inhibit cell division induced by TCR stimulation. Here, STAT3 GOF Tregs performed similarly to WT Tregs (Figure 3E).

We then tested the ability of STAT3 GOF T cells to upregulate Foxp3 in vitro and generate iTregs upon stimulation with anti-CD3, anti-CD28, TGF-β, and IL-2. Interestingly, STAT3 GOF T cells had a significantly reduced capacity to generate iTregs in vitro (Figure 3F). In vitro conversion was titratable with increasing doses of anti-CD3 in both WT and STAT3 GOF mice (Supplemental Figure 2F). At low concentrations of anti-CD3, where iTreg conversion was not optimized, WT and STAT3 GOF T cells had similar upregulation of Foxp3 (Supplemental Figure 2F). Overall, these mice, maintained in a specific pathogen–free facility, developed lymphoproliferation, but Treg enumeration, phenotype, and function was only minimally impacted, and they did not develop other clinical disease manifestations. A selective defect in iTreg generation was uncovered, and this reduced capacity to generate iTregs may be relevant for immune dysregulation under conditions that stress the system. Therefore, we next evaluated the Treg epigenetic and transcriptional signatures and in vivo function.

### STAT3 GOF and WT Tregs have a similar epigenetic and transcriptional profile.

Epigenetic regulation of the *Foxp3* locus is important for establishment of Treg stability and identity (24, 25). Hypomethylation of the Treg-specific demethylated region (TSDR), located ~4 kb downstream of the *Foxp3* promoter in the conserved noncoding sequence 2 (CNS2) of the *Foxp3* locus, is critical for stable and heritable Foxp3 expression (26). The impact of STAT3 GOF on TSDR methylation was measured by bisulfite sequencing using WT naive T cells, WT Treg, and STAT3 GOF Tregs isolated from the spleen and peripheral LNs of male mice. Cells from male mice were used due to random X-chromosome inactivation of the *Foxp3* gene in female mice.

**Figure 4:**
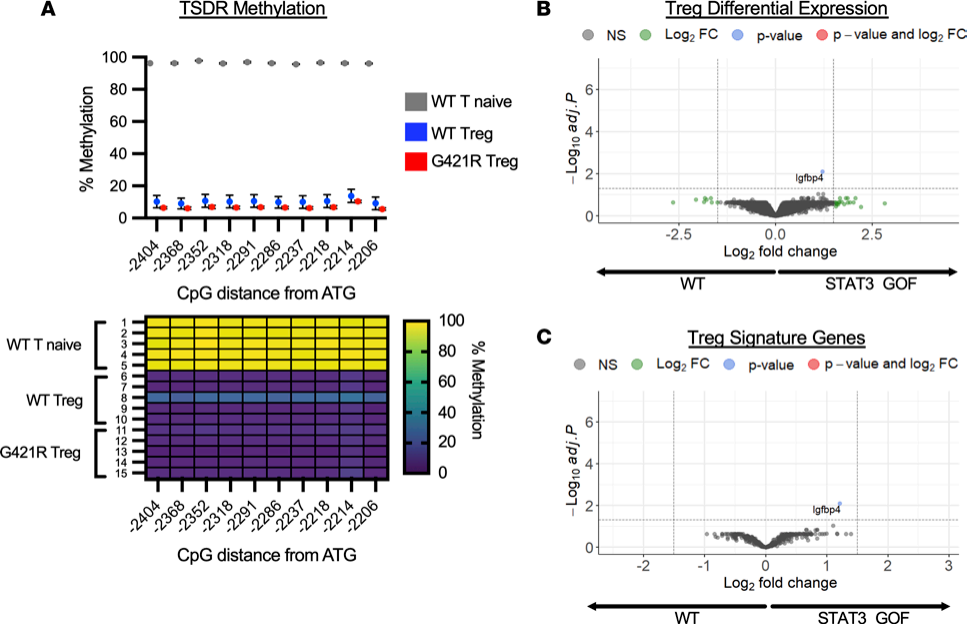
Epigenetic and transcriptional profile of Tregs. (**A**) Methylation status of 10 individual CpG motifs within the Treg-specific demethylated region (TSDR) in the *Foxp3* locus. Individual CpG motifs are numbered in reference to the translational start site (ATG). The average percent methylation is shown in the dot plot (top) for WT T naive cells, WT Treg, and G421R Treg isolated from the spleen and peripheral LN of adult mice (*n* = 5 for each group). Methylation patterns of each of the examined TSDR motifs of Tregs and naive T cells are shown in the heatmap (bottom). The color code ranges from purple (no methylation) to yellow (100% methylation). (**B**) Fold change versus *P* value (volcano) plot of gene expression in purified STAT3 GOF Tregs compared with WT Tregs (*n* = 4 samples for each genotype, with 3–5 pooled mice for each sample). Differential expression analysis was performed, and the results were filtered for only those genes with Benjamini-Hochberg FDR–adjusted *P* values less than or equal to 0.05. (**C**) Fold change versus *P* value (volcano) plot of gene expression in purified STAT3 GOF Tregs compared with WT Tregs of a subset of transcripts assigned to the Treg signature transcriptome. Transcripts with a log_2_ fold change > 1.5 or < –1.5 and *P* < 0.05 are considered significant.

A region of the CNS2 within the TSDR was amplified and sequenced by next-generation sequencing. Conversion of nonmethylated cytosine residues to uracil was achieved at an efficiency of > 99%. At any given CpG, the sequencing coverage ranged from 16,857 to 49,178 reads. As expected, WT naive T cells were highly methylated at the 10 CpG sites examined (ranging from 95% to 97% methylation). WT Tregs had low levels of methylation at the 10 CpG sites (methylation, 9%–13%), and this pattern was not different from Tregs isolated from STAT3 GOF mice (methylation, 5%–10%) (Figure 4A, top). Methylation signatures from individual mice overall were very similar (Figure 4A, bottom). Additional analysis of sorted Tregs from the thymus of WT and STAT3 GOF mice demonstrated similar levels of methylation in these populations (Supplemental Figure 3). At any given CpG, the sequencing coverage ranged from 32,318 to 69,152 reads. In the thymus, WT Tregs had intermediate levels of methylation at 10 CpG sites (methylation, 39%–67%), and this was similar to STAT3 GOF Tregs (methylation, 34%–67%). This degree of methylation is in line with what is reported in the literature for bulk thymic Tregs, and it implies that STAT3 GOF Tregs are able to undergo progressive demethylation with maturation (27).

Studies suggest that the Treg-specific transcriptional program is regulated by Foxp3 binding and that Foxp3 likely acts in conjunction with other cofactors, such as GATA3 and STAT3 (28–30). WT STAT3, in its activated phosphorylated state, has been shown to interact with Foxp3. Treg-specific expression of STAT3 is critical for control of pathogenic Th17 responses, and alterations in the Treg transcriptional program, including alterations in key chemokine receptors and suppressor molecules, may underlie this observation (31). To further investigate the impact of STAT3 GOF on Tregs, RNA-Seq was performed on sorted Foxp3^+^ Tregs from the spleen and LNs of WT and STAT3 GOF mice. Interestingly, *Igfbp4* was the only transcript with a significant difference based on adjusted *P* value, with a 1.2 log_2_ fold change between WT and STAT3 GOF Tregs for this transcript (Figure 4B). We specifically examined the differential expression of a set of 320 canonical Treg signature genes, and we found no significant differences (log_2_ fold change > 1.5 or < –1.5, adjusted *P* < 0.05) between WT and STAT3 GOF Tregs (Figure 4C) (28). Overall, under homeostatic conditions, there were not major differences observed in the transcriptome of STAT3 GOF Tregs.

### STAT3 GOF T cells adopt a Th1 phenotype, and iTregs are reduced in a disease model.

**Figure 5:**
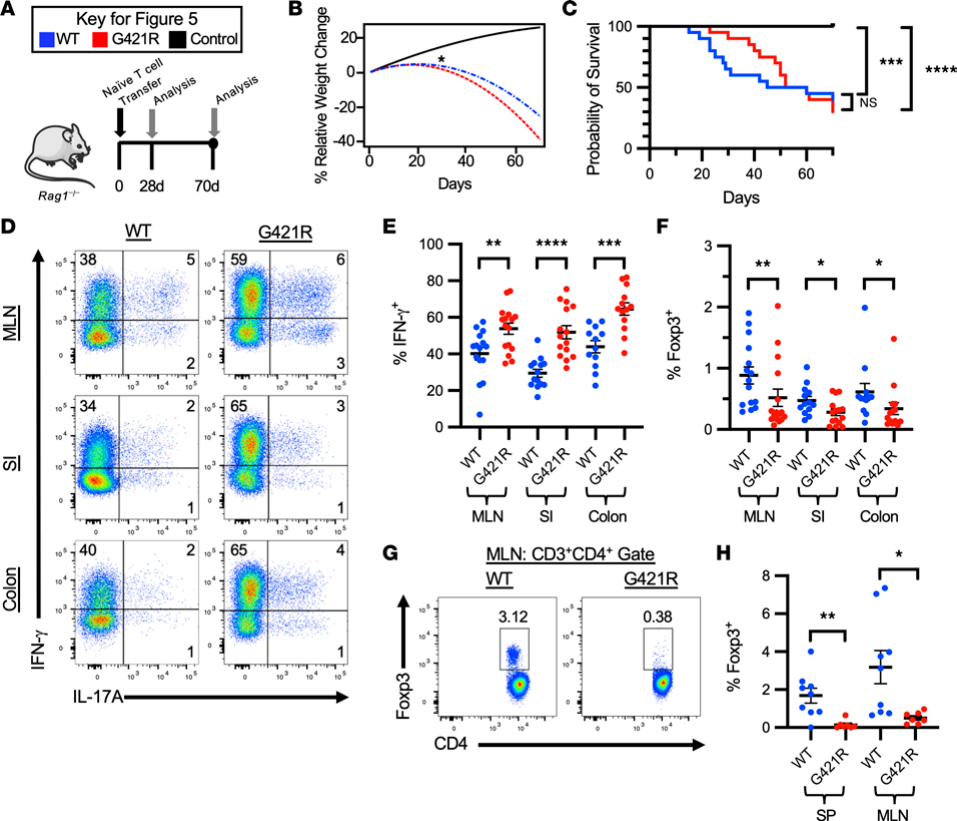
Th1 skewing and reduced pTreg generation in a colitis model. (**A**) Experimental design for establishment of the lymphopenia-induced colitis model. (**B**) Quadratic regression analysis modeling the percent relative weight change over time after the induction of experimental colitis with naive T cells isolated from WT (*n* = 20) or G421R mice (*n* = 20) compared with control C57BL/6 *Rag1*^–/–^ mice (*n* = 14). Asterisk represents day 28, at which a significant reduction in the weight was observed in colitis mice as compared with control mice. (**C**) Kaplan-Meier survival curves for the mice in **B**. Comparisons for the survival functions were done using the log-rank test. (**D**) Representative flow cytometry of CD3^+^CD4^+^ T cells isolated from the MLN, colon, and small intestine (SI) lamina propria lymphocytes and restimulated with PMA/ionomycin. Data were obtained at 28 days after the induction of experimental colitis. (**E**) Frequency of IFN-γ–producing CD3^+^CD4^+^ T cells in the indicated tissues, 28 days after induction of experimental colitis. (**F**) Percentage of in vivo–derived pTregs (CD3^+^CD4^+^ Foxp3^+^) in the indicated tissues, 28 days after the induction of experimental colitis. (**G**) Representative flow cytometry from the MLN showing pTreg induction in mice that survived to the conclusion of the experiment (70 days). (**H**) Frequency of pTregs (CD3^+^CD4^+^ Foxp3^+^) in the spleen and MLN of mice with WT or G421R colitis that survived to day 70. An unpaired *t* test was used for all comparisons with 2 groups, and Welch’s *t* test was used in the instance of unequal variance. *P < 0.05, **P < 0.01, ***P < 0.001, ****P < 0.0001.

A T cell transfer model of colitis was utilized to test T cell function in a disease setting. Naive CD4^+^ T cells (4 × 10^5^ CD4^+^EGFP^–^CD45RB^hi^ cells) were isolated by cell sorting from WT or STAT3 GOF mice and adoptively transferred into C57BL/6 *Rag1^–/–^* mice (Figure 5A). Weight loss and survival were similar between mice that received WT or STAT3 GOF T cells (Figure 5, B and C, and Supplemental Figure 4A). Mice that received either cell type had a significant reduction in weight by day 28 after transfer, as compared with control C57BL/6 *Rag1^–/–^* mice (Figure 5B). Therefore, initial analysis was focused on this early time point. Phenotypic analysis of transferred T cells after 28 days demonstrated an increased frequency of IFN-γ^+^ cells, but not IL-17A–producing cells, in the intestine lamina propria and MLNs of mice receiving STAT3 GOF T cells (Figure 5, D and E). Spleen, MLN, and intestinal lamina propria cell counts were similar at this early time point (Supplemental Figure 4B). Notably, after 28 days, mice with colitis induced by STAT3 GOF T cells had a significant reduction in the frequency of peripherally induced Tregs (pTregs) in the MLNs and intestinal lamina propria, suggesting decreased formation of pTregs in vivo, which was consistent with the in vitro data (Figure 5F). Strikingly, mice that received STAT3 GOF T cells and survived to the conclusion of the experiment (70 days), had an ~11-fold reduction in formation of in vivo–derived pTregs in the spleen and ~6-fold reduction of pTregs in the MLN (Figure 5, G and H). Total cell counts in the spleen were similar, but mice that received STAT3 GOF naive T cells had a decreased MLN total cell count (Supplemental Figure 4C). These results imply the surprising finding that STAT3 GOF T cells skew toward a Th1 phenotype, both in this disease model and in mice with germline STAT3 GOF. In addition, these data support the in vitro data, again demonstrating an impairment in pTreg induction.

### STAT3 GOF Tregs are functional in vivo.

**Figure 6:**
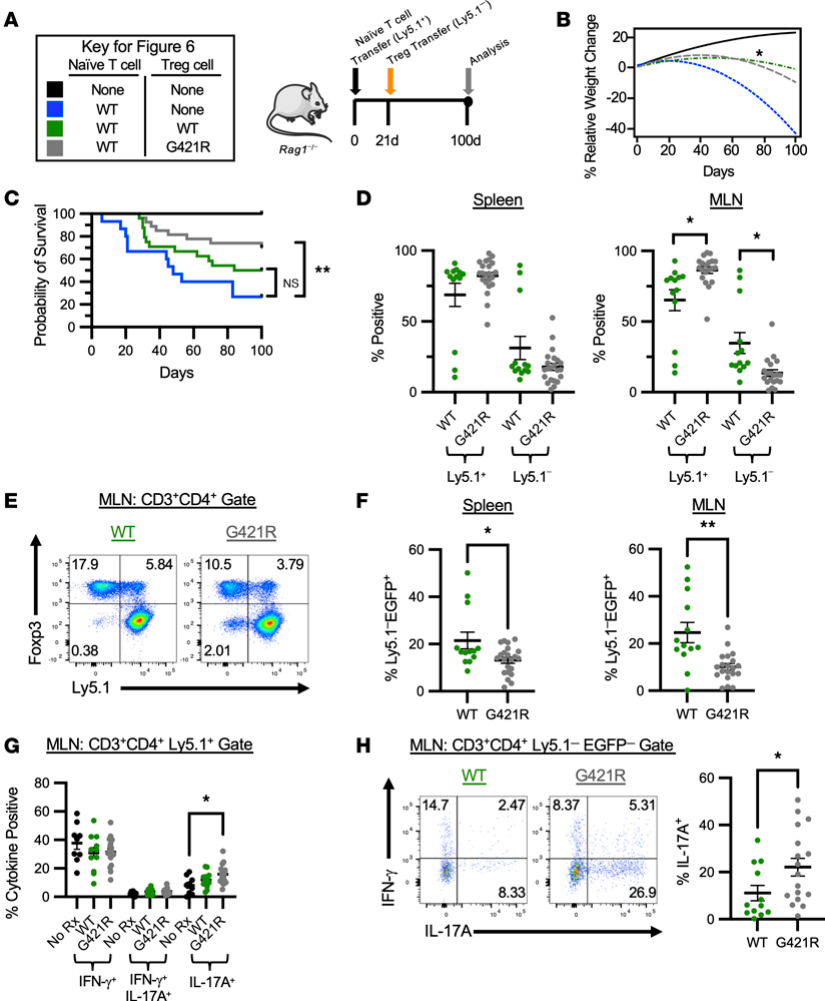
STAT3 GOF Tregs are functional in vivo. (**A**) Experimental design for the Treg treatment studies using the lymphopenia-induced colitis model. (**B**) Quadratic regression analysis modeling the percent relative weight change over time after the induction of experimental colitis with naive T cells isolated from WT mice. Mice were treated on day 21 with 1 × 10^6^ WT Tregs (*n* = 24) or G421R Tregs (*n* = 27), and weight patterns were compared with untreated mice (*n* = 15) or control C57BL/6 *Rag1*^–/–^ mice (*n* = 6). Asterisk represents day 77, at which a significant increase in the weight was observed in colitis mice treated with WT Tregs as compared with untreated mice. (**C**) Kaplan-Meier survival curves for the mice in **B**. Comparisons for the survival functions were done using the log-rank test. (**D**) Percentage of colitogenic Ly5.1^+^ T cells and Ly5.1^–^ T cells (treatment) isolated from the spleen and MLN of treated mice. (**E**) Representative flow cytometry from the MLN of treated mice showing Treg recovery. (**F**) Frequency of transferred Ly5.1^–^ WT or G421R Tregs recovered in the spleen and MLN of treated mice. (**G**) Frequency of MLN CD3^+^CD4^+^Ly5.1^+^ T cells that produce IFN-γ, both IFN-γ and IL-17A, or just IL-17A after stimulation with PMA/ionomycin. (**H**) Representative flow cytometry from the MLN of treated mice, demonstrating ex-Treg (CD3^+^CD4^+^Ly5.1^–^EGFP^–^) cytokine production after restimulation with PMA/ionomycin (left). Scatter plot demonstrating the frequency of MLN ex-Tregs (CD3^+^CD4^+^Ly5.1^–^EGFP^–^) that produce IL-17A (right). An unpaired *t* test was used for all comparisons with 2 groups, and Welch’s *t* test was used in the instance of unequal variance; and for those with 3 or more groups, 1-way ANOVA was used. *P < 0.05, **P < 0.01.

To establish the impact of STAT3 GOF on Treg function in vivo, we again utilized the T cell transfer model of colitis, as the role of Tregs in treating and preventing disease in this model has been well established (32, 33). There are some limitations of this model on the C57BL/6 background due to impaired pTreg and Th17 cell generation; however, murine genetic models are often created on the C57BL/6 background, and this colitis model remains useful for assessing Treg function in vivo (34). As demonstrated in the prior studies, by experimental day 28, colitis mice had significant weight loss compared with controls (Figure 5B). Therefore, mice with colitis induced by the transfer of 4 × 10^5^ WT CD4^+^Ly5.1^+^EGFP^–^CD45RB^hi^ cells were treated with either WT or STAT3 GOF Tregs on day 21. Mice were treated with 1 × 10^6^ Tregs that were isolated from either WT or STAT3 GOF mice (Ly5.2^+^) harboring the *Foxp3*^EGFP^ reporter to allow for sorting of a purified Treg population (Figure 6A). Weight change of individual mice within each experimental group was variable (Supplemental Figure 5A); however, pairwise comparison at the indicated time points demonstrated that mice treated with WT Tregs had improved weight gain compared with untreated mice, beginning at day 77 (*P* = 0.0473). Mice treated with STAT3 GOF Tregs had a trend toward improved weight gain by day 42, but the group comparisons did not reach significance (*P* = 0.0583) (Figure 6B). However, mice treated with STAT3 GOF Tregs actually had improved survival (Figure 6C). Spleen and MLN cell counts did not differ significantly between untreated and treated mice (Supplemental Figure 5B). The recovery of Ly5.1^+^ and Ly5.1^–^ marked cell populations was similar in the spleens of treated mice (Figure 6D). Conversely, in the MLN, there was an increased frequency of Ly5.1^+^ cells used to induce colitis and a corresponding decreased frequency of Ly5.1^–^ cells in mice treated with STAT3 GOF Tregs. The ratio of Ly5.1^+^/Ly5.1^–^ cells recovered in the MLN of treated mice was significantly higher in mice treated with STAT3 GOF Tregs compared with those treated with WT Tregs (14.9 versus 3.6, respectively), suggesting that local control of colitis cell accumulation may be impaired in these mice (Figure 6D).

Indeed, the frequency of Tregs found in the spleen and MLN of mice treated with STAT3 GOF Tregs was reduced compared with mice treated with WT Tregs (Figure 6, E and F). However, this did not correlate with an increased frequency of ex-Tregs, defined as cells that were Ly5.1^–^EGFP^–^ (Supplemental Figure 5C). Interestingly, the frequency of MLN Ly5.1 cells producing IL-17A was increased in mice treated with STAT3 GOF Tregs, as compared with untreated mice (Figure 6G). In the spleen, IL-17A^+^Ly5.1^+^ cells were increased in both groups of treated mice compared with untreated mice (Supplemental Figure 5D). Although the frequency of ex-Tregs was similar in mice treated with WT or STAT3 GOF Tregs, a higher frequency of STAT3 GOF ex-Tregs produced IL-17A (Figure 6H).

In summary, STAT3 GOF Tregs improved survival in a mouse model of experimental colitis, despite impaired Treg recovery and control of Ly5.1^+^ T cell accumulation and IL-17A secretion. Furthermore, ex-Tregs from STAT3 GOF mice demonstrated dysregulated production of IL-17A. This suggests that Treg function may not be normal in situations where the system is stressed.

### Single-cell RNA-Seq of STAT3 GOF patient T cells.

To explore the dysregulated T cell phenotype in human STAT3 GOF syndrome, T cells were isolated from the peripheral blood of 3 healthy age- and sex-matched donors (C1–C3) and 3 patients with STAT3 GOF syndrome (patients 1–3 [P1–P3]). Cells were rested in media or stimulated with anti-CD3/CD28 (C1s–C3s, P1s–P3s) for 16 hours and then subjected to single-cell RNA-Seq analysis. Human STAT3 variants were located throughout the protein and included variants in the DNA-binding domain p.T389S (c.1165A>T) in P1, the NT domain p.R70H (c.209G>A) in P2, and the coiled-coil domain p.F174S (c.521T>C) in P3. Additional details regarding the patients can be found in Supplemental Table 1. All patients were on immunosuppressive therapy, P1 and P2 were stable, but P3 had progressive disease, and samples were obtained at a single point in time; these are limitations for interpretation of the data.

**Figure 7:**
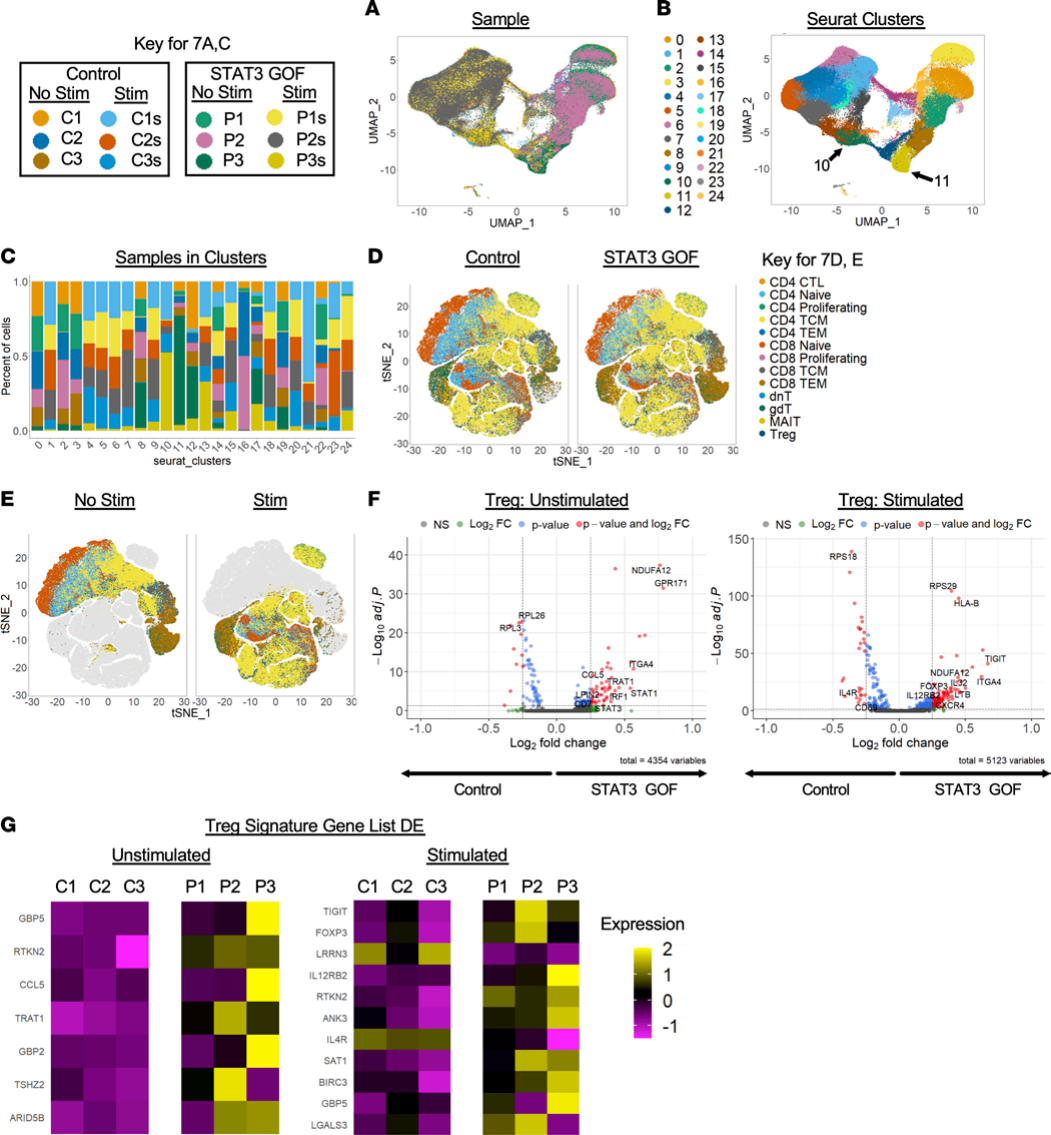
STAT3 GOF syndrome T cell single-cell RNA-Seq. Control and patient T cells were isolated from PBMCs by CD3 negative selection; they were then incubated in media alone (no stimulation) or with anti-CD3/CD28 stimulation for 16 hours before submitting for single-cell RNA-Seq analysis. (**A** and **B**) Unsupervised dimensionality reduction analysis of single-cell RNA-Seq transcriptome data from STAT3 GOF and healthy controls showing sample identity (**A**) and unique clusters (**B**). (**C**) Cluster composition as defined by sample identity. (**D** and **E**) Identification of cells using Azimuth cell prediction program with t-distributed stochastic neighbor embedding (t-SNE) plot showing T cells split based on the affected status (**D**) or by stimulation status (**E**). (**F**) Volcano plot showing differential expression (adjusted *P* < 0.05, average log_2_ fold change > 0.25 or < –0.25) in cells identified as Tregs, comparing cells from unstimulated (left) or stimulated conditions (right). (**G**) Heatmap showing the average log_2_ fold change of differentially expressed genes found in the Treg signature gene list for control and STAT3 GOF Tregs.

Unsupervised dimensionality reduction analysis of single-cell RNA-Seq transcriptome data from STAT3 GOF and healthy controls identified 24 unique clusters (Figure 7, A and B). After the initial filtering steps, 140,931 cells were analyzed. Clusters 8, 11, 17, and 24 were enriched (at least 60% of the cells) for cells from STAT3 GOF patients (Figure 7C and Supplemental Figure 6A). Clusters 10 and 11 were dominated by cells from patient samples from P3 or P3s (>50% of cells in the cluster) (Figure 7C and Supplemental FIgure 6B).

Cell populations were further identified by their expression of canonical cell markers using the Azimuth human PBMC reference to predict cell types (NIH Human Biomolecular Atlas Project [HuBMAP]) (35). All cells predicted as T cells (total of 139,421 cells) were segregated according to affected status, as well as on the presence or absence of stimulation (Figure 7, D and E). Tregs were identified based on expression of *RTKN2*, *FOXP3*, *AC133644.2*, *CD4*, *IL2RA*, *TIGIT*, *CTLA4*, *FCRL3*, *LAIR2*, and *IKZF2*. Tregs dominated clusters 18, 19, and 20 (Supplemental Figure 6C). Differential expression analysis of all cells classified as Tregs revealed 59 genes differentially expressed between control and STAT3 GOF patients in the unstimulated samples and 112 genes with significant differential expression in the stimulated samples (Figure 7F). In the unstimulated Tregs, genes overexpressed in STAT3 GOF patients included *NDUFA12*, *GPR171*, *ITGA4*, *TNFRSF13B*, *TRAT1*, *LPIN2*, and *CD7* and also, primarily in patient sample P3, *CCL5*, *IRF1*, *STAT1*, and *STAT3* (Figure 7F, left). In the stimulated Tregs, there was similarly increased expression of *NDUFA12*, *ITGA4*, and *TNFRS13B*, but there was also increased expression of *TIGIT*, *IL32*, *FOXP3*, *LTB*, *CXCR4*, and *IL12RB2* in STAT3 GOF samples and decreased *CD69* and *IL4R* (Figure 7F, right). Overall, small differences in the average log_2_ fold change were observed, suggesting that the circulating Tregs in control and patient samples were similar.

Among “Treg signature” genes identified in a published data set (36), 322 of 367 genes (total of 386 probe sets) were found in our data set. There were only significant differences in the expression of 7 transcripts, including *GBP5*, *RTKN2*, *CCL5*, *TRAT1*, *GBP2*, *TSHZ2*, and *ARID5B*. Expression differences noted for *CCL5* and *GBP5* were primarily due to changes seen in sample P3, and alterations in *TSHZ2* expression were due to sample P2 (Figure 7G, left). Expression of the “Treg Signature” was analyzed within the stimulated Treg subset, with 11 differentially expressed transcripts identified, including *TIGIT*, *FOXP3*, *LRRN3*, *IL12RB2*, *RTKN2*, *ANK3*, *IL4R*, *SAT1*, *BIRC3*, *GBP5*, and *LGALS3* (Figure 7G, right).

**Figure 8:**
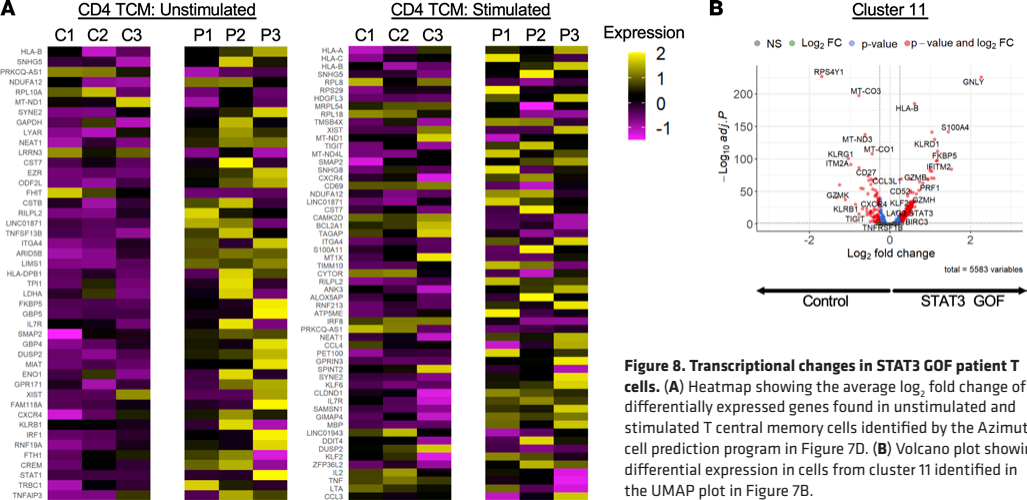
Transcriptional changes in STAT3 GOF patient T cells. (**A**) Heatmap showing the average log_2_ fold change of differentially expressed genes found in unstimulated and stimulated T central memory cells identified by the Azimuth cell prediction program in Figure 7D. (**B**) Volcano plot showing differential expression in cells from cluster 11 identified in the UMAP plot in Figure 7B.

We further examined the CD4 T central memory (TCM) subset (defined by expression of *IL7R*, *TMSB10*, *CD4*, *ITGB1*, *LTB*, *TRAC*, *AQP3*, *LDHB*, *IL32*, and *MAL*) because this was heavily represented in multiple clusters. Forty-five unique transcripts were differentially expressed between unstimulated control and STAT3 GOF CD4 TCM cells genes (adjusted *P* < 0.05, average log_2_ fold change > 0.25 or < –0.25). Transcripts identified as overexpressed in STAT3 GOF again included *TNFSF13B* and *ITGA4*, as well as *ARID5B*, *LIMS1*, and *TNFAIP3* and, in sample P3, *STAT1* and *IRF1* (Figure 8A). Examination of enriched ontology clusters found significant enrichment in several pathways, with cellular response to cytokine stimulus, aerobic glycolysis, and IFN-γ signaling among those pathways with the most significant *P* values (Supplemental Figure 6D).

In stimulated CD4 TCM cells, there were 57 differentially expressed transcripts (adjusted *P* < 0.05, average log_2_ fold change > 0.25 or < –0.25). Transcripts identified as overexpressed in STAT3 GOF included *TIGIT*, *CXCR4*, *ITGA4*, *KLF6*, and *IL7R*. Transcripts underexpressed in STAT3 GOF patients compared with controls included *IL2*, *TNF*, *LTA*, *CD69*, *IRF8*, and *BCL2A1* (Figure 8A, right). Enriched ontology cluster analysis performed with genes overexpressed in stimulated TCM cells from STAT3 GOF patients demonstrated enriched terms for antigen processing and presentation of endogenous peptide antigen via MHC-I, negative regulation of immune system process, and regulation of cell-to-cell adhesion (Supplemental Figure 6E).

Clusters that were dominated by cells from samples P3/P3s were largely identified as CD8 T cells, including CD8 TCM and CD8 TEM cells, as well as γδ T cells. For example, cluster 10, a cluster made up of stimulated cells, largely consists of cells from sample P3s. These cells are identified as CD8 TEM and γδ T cells by the Azimuth program. Cluster 11 is largely dominated by unstimulated cells, particularly from patient sample P3 (~73% of cells). This cluster is identified primarily as CD8 TEM cells (~95% of cells). The CD8 TEM cells were identified based on expression of transcripts for *CCL5*, *GZMH*, *CD8A*, *TRAC*, *KLRD1*, *NKG7*, *GZMK*, *CST7*, *CD8B*, and *TRGC2*. Here, differential expression analysis revealed increased expression of 245 genes and decreased expression of 97 genes in STAT3 GOF compared with controls (Figure 8B). Those genes noted to have increased expression included *GNLY*, *GZMH*, *PRF1*, *GZMB*, *KLRD1*, *CX3CR1*, and *STAT3*. Gene list analysis of those genes upregulated in cluster 11 STAT3 GOF cells identified enriched ontology clusters, including cytokine signaling in immune system and cell activation, but also demonstrated positive regulation of the immune response and cytokine production, as well as the IL-12 pathway (Supplemental Figure 6F). Accordingly, there was also a relative decrease in clusters that were identified as naive T cells, including cluster 0 (mostly naive CD4 T cells), cluster 1 (stimulated CD4 naive and TCM cells), and clusters 3 and 6 (naive CD8 T cells).

In summary, the expression of canonical Treg genes in healthy control Tregs and Tregs from treated patients with STAT3 GOF syndrome were similar, with only small differences in average log_2_ fold change observed in a few select genes. These data support the patterns seen in the murine data, and they suggest that STAT3 GOF confers a mild impact on circulating Tregs and on the composition and transcriptomic profile of circulating T cells.

## Discussion

In this study, we generated a mouse model of a human STAT3 GOF variant to study the impact of STAT3 GOF on T cells, with a focus on Tregs. We demonstrate that CD4 and CD8 T cell dysregulation commences early in development, culminating in lymphoproliferative disease and Th1-dominated CD4^+^ T cell skewing. However, Treg numbers, function, and phenotype were only mildly impacted. Specifically, we observed an accumulation of Tregs paralleling the lymphoproliferation, as well as a defect in the generation of iTregs. Lastly, we studied the immune phenotype of patients with STAT3 GOF syndrome, also demonstrating a mild Treg impact by single-cell RNA-Seq, as well as an expanded CD8^+^ TEM population.

Although a reduction in Treg numbers and/or function has been implicated as an etiology of autoimmunity, increased numbers of Tregs have also been observed in several autoimmune diseases, suggesting that, in some instances, Treg expansion and/or induction may actually constrain disease progression (37, 38). Indeed, in other models of organ-specific autoimmunity, partial Treg depletion promotes disease progression and death, supporting the importance of Treg presence at sites of inflammation (39). Data on human Tregs in disease are largely limited to peripheral blood specimens, as studying local Treg generation and enumeration in patients is often not feasible. The impact of chronic inflammation on Treg phenotype, function, and deviation from an antiinflammatory role remains under investigation (40). A Treg defect has been implicated in the pathogenesis of STAT3 GOF syndrome based on the clinical phenotype of patients with active disease, and with peripheral blood specimens demonstrating reduced Treg numbers or reduced CD25 expression and, in a handful of patients, reduced Treg function (10, 15). Our data do not support the hypothesis that inherent defects in Tregs are the main driver of disease — rather, they align with a recently published murine model of a diabetogenic STAT3 GOF variant (41). In this study, a knock-in mouse model on the nonobese diabetic background was created using a STAT3 GOF DNA-binding domain variant (p.K392R) (41). While Treg generation was impaired in vitro, functional analysis of Tregs in an antigen-specific system did not implicate Tregs as key drivers of disease. Rather, this group identified a clonally expanded, pathogenic CD8 effector T cell population and CD8 T cell dysregulation as a key component of type 1 diabetes development. We also identified an increased frequency of activated, effector CD8 T cells in murine secondary lymphoid organs. Furthermore, our single-cell RNA-Seq data from STAT3 GOF patients demonstrated an expanded cluster in 1 patient that was composed primarily of CD8 TEM cells, with increased expression of several CD8 cytotoxic and effector transcripts compared with control samples. Accordingly, preclinical models examining underlying disease mechanisms are essential to understanding disease pathogenesis and treatment strategies in rare diseases such as IEI.

Precision medicine for IEI — for example, with small molecules or biologics — presents a challenge, as the rarity of these disorders makes it difficult to perform controlled trials. Therefore, in-depth knowledge of underlying immunological mechanisms of disease is needed to help guide clinical decision making and inform on the utility of repurposing currently available medications for IEIs. STAT3 is a highly conserved protein, with only 1 amino acid different between mice and humans (a tolerated substitution of aspartic acid for glutamic acid at the C terminus; Uniprot.org), suggesting that the mouse is a good model organism to study STAT3-mediated disease. Indeed, a mouse model of *STAT3* loss of function (hyper IgE syndrome) recapitulated many aspects of disease, including impaired Th17 cells, and mouse models of IPEX and APECED have proved useful as tools for studying T cell tolerance (13, 42, 43). Autosomal dominant PIRDs, like STAT3 GOF, demonstrate profound clinical heterogeneity, and there are likely additional factors contributing to disease (such as genetic or environmental) that are not readily apparent in a controlled, inbred animal model.

There is ongoing interest and clinical efforts to harness the therapeutic potential of Tregs for the treatment of autoimmunity and IEI (44–46). Particularly in rare IEI with immune dysregulation, it is critical to understand whether a defect in Treg generation or function exists, prior to proceeding with Treg-targeted therapies. Furthermore, the specific strategy sought to enhance Tregs will likely vary based on the underlying defect. For example, strategies that focus on the expansion of polyclonal Tregs may be favored over strategies that promote the generation of pTreg or iTregs in conditions in which a defect in iTreg production exists. While secondary lymphoid and blood Treg numbers and function were largely normal or even increased in STAT3 GOF mice, we observed a defect in the in vitro and in vivo generation of iTreg and pTregs in STAT3 GOF mice. In patients with STAT3 GOF syndrome, defects in peripheral blood Treg numbers are variably observed (47). Perhaps this may also account for some variability in the response to treatment. These data may also support the hypothesis that, under conditions of immune homeostasis, STAT3 GOF Tregs are functional and play an important role in limiting disease progression, but — under conditions of stress, such as infection or active inflammation, or in cases where other environmental or genetic factors exist — pTreg generation may not be sufficient to constrain dysregulated immune responses. This may, in part, contribute to the deficiency in peripheral blood Treg numbers seen in STAT3 GOF patients. Indeed, attempts to dissect the human Treg population based on cell surface markers have demonstrated the considerable heterogeneity of this compartment, which likely also fluctuates during disease states (38, 48, 49).

The STAT3 pathway is activated downstream of numerous cytokines and growth factors, and it has been broadly implicated in both innate and adaptive immune pathways (50). Studies have demonstrated that germline STAT3 GOF variants lead to alterations in human monocyte and DC populations (51). Changes in relevant antigen presenting cell phenotype and chemokine expression may have an impact on local pTreg induction and/or localization (34, 52). Prior work has demonstrated that, although Treg subsets utilize similar mechanisms, unique TCR repertoires (and likely, specificity) support the notion that both peripheral and thymic-derived Tregs are important to enforce tolerance (23, 53, 54). CD4^+^ T cells poised to adopt a pTreg fate also help divert cells away from other Th states present in the local niche, and this is best modeled by the Treg/Th17 paradigm (55). Interestingly, in the murine colitis model, we demonstrate that an increased frequency of STAT3 GOF Tregs that had downregulated Foxp3 expression became IL-17A–producing ex-Tregs. In summary, these data suggest that Tregs are less likely to be a useful primary therapeutic target for STAT3 GOF syndrome.

In our murine model on the C57BL/6 background, we did not observe spontaneous endocrinopathy, cytopenias, or other organ-specific autoimmunity such as enteropathy. It is likely that other genetic modifiers and/or environmental exposures contribute to the variability of the phenotypes that are observed in STAT3 GOF syndrome, both in mice and humans. Surprisingly, despite the important role of STAT3 in Th17 cell generation, we did not find Th17-skewing in vivo. Rather, we observed increased IFN-γ production and Th1-skewing in the dysregulated T cells, a phenotype not typically reported to be associated with STAT3 activity. A prior study demonstrated that ex vivo stimulation of T cells from a patient with the STAT3 p.K392R variant led to increased IFN-γ and TNF-α production (21); however, there is very little known about Th cell polarization and function in STAT3 GOF. STAT3 usually forms homodimers but can also form heterodimers with STAT1 (56). For example, STAT1 serves an important role in shaping the unique IL-27 cytokine signature (56), a cytokine that promotes early Th1 cell commitment (57, 58). It is possible that variants in STAT3 may confer alterations in STAT3 homo/heterodimer formation and DNA-binding specificities, which can impact downstream transcriptomic profiles. Polarization of cells to a Th1-predominant phenotype may have implications for therapy in these patients and would support a broader cytokine-directed approach with jakinibs or even consideration of drugs targeting type I cytokines/signaling, over a cytokine-specific therapy, such as anti–IL-6 therapy (59). Naive T cells from STAT3 GOF mice did not demonstrate increased Th1 polarization in vitro, which suggests that this is an in vivo phenomenon. Whether this is an antigen-driven or independent process requires further investigation. Memory phenotype CD4^+^ T cells have been described as a subset of cells with high expression of CD44 that are highly proliferative in the steady state and can develop in the absence of foreign antigen recognition (60). These cells have been shown to adopt an innate Th1-type effector phenotype (61). Here, we have demonstrated an increased frequency of CD4^+^CD44^hi^Ki-67^+^ T cells in the STAT3 GOF mice. It will be important to explore this further, including an investigation of these memory phenotype T cells in humans and autoimmune disease (62).

STAT3 also has an important role in T follicular helper (Tfh) and T follicular regulatory cell biology, and this may contribute to the apparent immune dysregulation (63). Some monogenic IEIs result in changes in the number and phenotype of circulating Tfh cells (64). An increased frequency of circulating Tfh1 cells has been observed in the peripheral blood of STAT3 GOF patients; this is a subset of Tfh cells that express defining features of Th1 cells and produce IFN-γ (65). The role of circulating Tfh1 cells in STAT3 GOF is still under investigation. It is possible that an exuberant and dysregulated circulating Tfh1 population may be contributing to autoimmunity in these cases, by enhancing the development of T-bet^+^ B cells and humoral B cell responses that dominate in an IFN-γ–governed environment, leading to a skewed Th1-type response (66). Additional studies are needed to clarify the role of these cell types in the establishment of immune dysregulation. Overall, our studies support a dysregulated T effector phenotype as a key driver of disease, rather than a primary imbalance or ineffectiveness of the Treg response. These results also highlight the importance of preclinical models to investigate disease mechanisms to help guide therapeutic approaches in rare diseases.

## Methods

Supplemental Methods are available online with this article.

### Generation of STAT3 GOF mice.

*Stat3*^p.G421R/+^ (referred to as G421R or STAT3 GOF) mice were generated by the Hope Center Transgenic Vectors Core at Washington University School of Medicine using CRISPR-Cas9 technology (67). Mice were backcrossed to C57BL/6 (Charles River Laboratories) using a speed congenic approach to ensure that they were fully backcrossed.

### Mice.

*Foxp3*^EGFP^ mice were obtained from The Jackson Laboratory (stock no. 006772) and screened, per manufacturer protocol (23). *Foxp3*^EGFP^ mice were crossed to STAT3 GOF mice and NCI B6-Ly5.1/Cr congenic mice (Charles River Laboratories). *Rag1*^–/–^ mice were obtained from The Jackson Laboratory (stock no. 034159). Mice were used between 3 and 30 weeks of age, as indicated in the figures. For most studies, unless indicated, male and female mice were used between 7 and 16 weeks of age.

### Cell purification and adoptive transfer.

Splenocytes with or without pooled peripheral LNs (axillary, brachial, inguinal) were isolated, and CD4^+^ T cells were purified with the MojoSort mouse CD4 T cell isolation kit (catalog 480005); they were then stained with anti-CD4 (clone RM4-5, BD Biosciences, 558107) plus anti-CD45RB (clone 16A, BD Biosciences, 562848) anti-murine antibodies, and sorted on the basis of antibody and EGFP fluorescence. Sorting was done on a BD FACS AriaFusion flow cytometer (BD Biosciences). Colitis was induced in 6- to 8-week-old *Rag1*^–/–^ mice by i.p. injection of 4 × 10^5^ CD4^+^Ly5.1^+^EGFP^−^CD45RB^hi^ cells isolated from WT or STAT3 GOF mice. In some experiments, mice were treated 21 days after the induction of colitis with 1 × 10^6^ CD4^+^Ly5.2^+^EGFP^+^ WT or STAT3 GOF Tregs. Mice were weighed at least twice weekly and sacrificed when moribund or at the conclusion of the experiment. Mechanistic details for the lamina propria digest and isolation of lymphocytes are provided in the Supplemental Methods.

### BM chimeras.

BM chimeras were generated by transplanting whole BM cells from Ly5.2^+^ donors into Ly5.1^+^ recipients that were irradiated with 2 doses of 550 cGy. In total, 2 × 10^6^ cells were injected retro-orbitally into lethally irradiated recipients. Recipients received 0.5 mg/mL sulfamethoxazole and 0.1 mg/mL trimethoprim in drinking water ad libitum for 2 weeks after transplant. Cells were allowed to engraft in recipients for 12 weeks before analysis.

### Treg in vitro conversion.

Sorted CD4^+^EGFP^–^CD45RB^hi^ naive T cells from *Foxp3*^EGFP^ and *Stat3*^p.G421R/+^ *Foxp3*^EGFP^ mice (1 × 10^6^/mL) were cultured in R10 media (RPMI 1640 [Corning] supplemented with 10% FBS [MilliporeSigma], 1% penicillin-streptomycin [MilliporeSigma], 1% L-glutamine [MilliporeSigma]), with anti-CD3 mAb–coated (clone 145-2C11 at 10 μg/mL, Biocell, catalog BE0001-1) dishes in the presence of soluble anti-CD28 mAb (2 μg/mL; clone 37.51, Biocell, catalog BE0015-1), TGF-β1 (5 ng/mL; Cell Signaling Technology, catalog 5231), and 20 ng/mL IL-2 (PeproTech, catalog 212-12). In some experiments, the amounts of anti-CD3 and anti-CD28 were titrated (as indicated, anti-CD3 5 μg/mL and 2.5 μg/mL; anti-CD28 1 μg/mL). After 72 hours, cells were analyzed by flow cytometry.

### Cell stimulation for phospho-STAT3 analysis.

Splenic lymphocytes were isolated and cultured at 1 × 10^6^ cells/mL in R10 media, with or without 10 ng/mL recombinant murine IL-6 (Peprotech), for 15 minutes at 37°C with 5% CO_2_. Cells were then washed with R10 and returned to 37°C for the remaining incubation time (90 minutes).

### Antibodies and flow cytometry.

Cells were collected from the thymus, spleen, MLN, peripheral LNs, colon, and small intestine and were stained as indicated. The anti-mouse antibodies are detailed in Supplemental Table 2. Cells were washed with staining buffer (1× PBS containing 2% FBS and 1 mM EDTA [Thermo Fisher Scientific]), treated with Fc block, stained with surface antibodies for 30 minutes at 4°C, and then washed with staining buffer. Intracellular cytokine staining was performed after a 4-hour restimulation with PMA (5ng/mL; Sigma-Aldrich, catalog P1585) and ionomycin (0.5uM; Sigma-Aldrich, catalog I0634-1MG) in the presence of brefeldin A (1 μL/mL; BD Biosciences). Details on phospho-flow cytometry and intracellular staining are in the Supplemental Methods. Data were acquired on an LSRFortessa (BD Biosciences) or Cytek Aurora. Data were analyzed using FlowJo 10.7.1.

### Treg suppression assay.

Splenocytes from *Rag1*^–/–^ mice were isolated, plated, and incubated with variable numbers of sorted WT or STAT3 GOF Tregs (CD4^+^EGFP^+^) and Tag-it Violet–labeled [BioLegend] WT CD4^+^EGFP^–^CD45RB^hi^ naive T cells in the presence of anti-CD3 (1 μg/mL). Cells were incubated at 37°C for 72 hours, and proliferation was assessed by flow cytometry. See Supplemental Methods for additional details.

### Bulk RNA-Seq.

Cell sorting for Tregs was performed as described above. Tregs from the spleen and LNs of WT or STAT3 GOF mice were pooled (3–5 mice per sample), and 4 samples for each genotype were sequenced. RNA was extracted using the RNeasy Mini Kit (Qiagen, catalog 74014). Bulk RNA-Seq was performed by the Genome Technology Access Center at the McDonnell Genome Institute at Washington University School of Medicine using a polyA-based amplification approach with the Takara-Clontech SMARTer low input RNA kit. See Supplemental Methods for details on processing and sequencing analysis.

### Bisulfite conversion and methylation analysis.

For methylation analysis, cells from male mice were used due to random X-chromosome inactivation of the *Foxp3* gene in female mice. Genomic DNA was isolated from WT and STAT3 GOF EGFP^+^ Treg and WT CD4^+^EGFP^–^CD45RB^hi^ naive T cells sorted by FACS, according to the manufacturer’s directions (Quick-DNA MiniPrep or MicroPrep, catalog D3024 or D3020). Amplicon bisulfite sequencing was performed similar as in McDonald et al. (68). Additional details on the primers, DNA libraries, sequencing, and analysis are provided in the Supplemental Methods.

### Single-cell RNA-Seq.

Blood samples were obtained from 6 individuals: 3 patients with STAT3 GOF syndrome and 3 age-matched healthy controls. Peripheral blood mononuclear cells (PBMCs) were isolated using Ficoll-Paque Plus (Cytiva, catalog 17-1440-02) density gradient centrifugation (872*g*, 20 minutes at room temperature), subjected to RBC lysis, and frozen at –80°C before transferring for storage in liquid nitrogen. On the day of the experiment, PBMCs were thawed at 37°C, incubated in DNase I solution [Roche], filtered, and then isolated using the EasySep Human T cell Isolation Kit (Stemcell Technologies, catalog 17951) following the manufacturer’s instructions. Cells were seeded at 1 × 10^6^ cells/mL in R10 media and placed in a 37°C incubator with or without stimulation. Cell stimulation was performed with 25 μL/mL immunocult human CD3/CD28 activator reagent (Stemcell Technologies, catalog 10971) for 16 hours. Cells were filtered, washed with PBS/2% FBS/1 mM EDTA, and resuspended in PBS/0.04% BSA solution [MilliporeSigma] for analysis. Samples were further processed by the Genome Technology Access Center at the McDonnell Genome Institute at Washington University School of Medicine, where single-cell suspensions were loaded onto a Chromium Single Cell Chip (10X Genomics) according to the manufacturer’s instructions.

### Single-cell RNA-Seq data processing.

Single-cell raw data was filtered, aligned, and aggregated using Cellranger v6.0.0 (count and aggr functions) (69). The feature-barcode matrix analysis was performed using the R package Seurat v4 (35). In total, 147,052 cells were present in the assay. Cells with more than 15% mithochondrial RNA and fewer than 200 expressed features were removed, along with cells with more than 4,000 features (unstimulated cells) or 6,000 features (stimulated cells); 140,931 cells remained after filtering. Data matrices were split into lists based on affected status and normalized using SCTransform. Samples were integrated based on the expression of 3,000 features; data integration was done using reciprocal principal component analysis (RPCA) with 20 k.anchors (how many neighbors [k] to use when picking anchors) and 30 dimensions for the reduction (70). Cell types were predicted using Azimuth’s human PBMC data (celltype.l2 gene list) (70). Cells classified as anything other than T cell subsets were filtered out, and with that, 139,421 cells remained. For differential expression between STAT3 GOF and control samples, the data were log normalized (scale = 10,000) to get an ideal difference between genes that are lowly expressed, and the Seurat’s “FindVariableFeatures” function with default parameters was used to calculate average expression. Heatmaps of statistically significant differentially expressed genes (adjusted *P* < 0.05, log_2_ fold change > 0.25 or < –0.25) were generated using bulk average RNA expression of normalized counts with the AverageExpression() function in R for each condition. Differential expression gene lists were generated based on the nonparametric Wilcoxon rank-sum test. Pathway analysis was performed using Metascape (71). Only genes with adjusted *P* < 0.05 were retained for pathway analysis. Figures were generated using Seurat, dittoseq, and EnhancedVolcano R packages (https://github.com/kevinblighe/EnhancedVolcano; commit ID, 7abca28) (35, 70). Data have been deposited under accession no. GSE207936 (https://www.ncbi.nlm.nih.gov/geo/query/acc.cgi?acc=GSE207936).

### Statistics.

Statistics were calculated using Prism 9. In general, a 2-tailed unpaired *t* test was used for all comparisons with 2 groups, and Welch’s *t* test was used in the instance of unequal variance. For comparisons with 3 or more groups, samples were analyzed with a 1-way ANOVA (with Tukey’s multiple-comparison test) or a 2-way Welch ANOVA if the variance was unequal (with Dunnett’s multiple-comparison test). In instances with 2 variables, 2-way ANOVA was performed. The comparisons between groups for overall survival functions were done using the log-rank test. We contrasted weight-change profiles between groups using a random coefficient model with quadratic day terms and group interaction with the quadratic day terms (SAS 9.4 mixed procedure) (72). Degrees of freedom were adjusted with the Kenward-Roger method for bias correction.

### Study approval.

Animal studies were approved by the IACUC at Washington University. Human blood samples were sourced ethically, and research use was in accordance with the terms of the informed consent under IRB-approved protocols at the authors’ institutions (Washington University School of Medicine and Johns Hopkins All Children’s Hospital).

## Author contributions

EGS and MAC designed the work and wrote the manuscript. EGS, KAT, SIR, NS, ZJG, and RJFB performed experiments. EGS, KAT, AK, JRE, and MAC analyzed and interpreted the data. TPV designed and performed experiments. JWL, JJB, and AT provided patient samples and contributed to the manuscript. LGS supervised experiments.

## Supplementary Material

Supplemental data

## Version 1 09/22/2022

In-Press Preview

## Version 2 11/08/2022

Electronic publication
