# Supplementary material for: A human STAT3 gain-of-function variant confers T cell dysregulation without predominant Treg dysfunction in mice
Source: JCI Insight. 2022 Nov 8;7(21):e162695. doi: 10.1172/jci.insight.162695 (PMC9675480; doi:10.1172/jci.insight.162695)
Supplement: Supplemental data [file jciinsight-7-162695-s283.pdf]

A human STAT3 gain-of-function variant confers T cell dysregulation without predominant Treg dysfunction in mice

Schmitt EG, et al.

Contents:

Supplemental Figures 1-6
Supplemental Table 1
Supplemental Methods
Supplemental Table 2
Supplemental References

Supplemental Figure 1

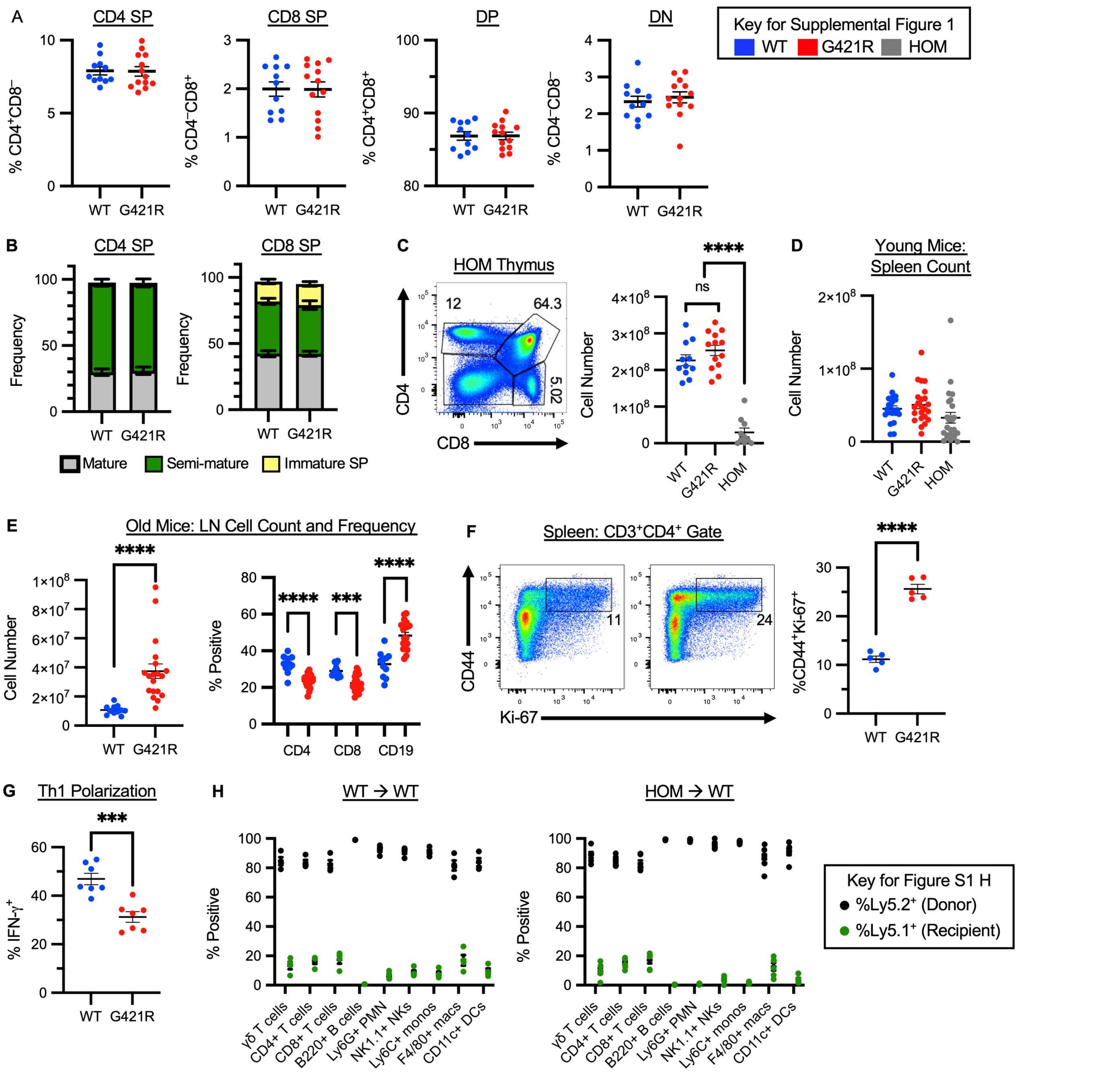

Supplemental Figure 1. Characterization of the STAT3 GOF mice. (A) Frequency of CD4 single positive (SP), CD8 SP, CD4 CD8 double positive (DP) and CD4 CD8 double negative (DN) T cells in the thymus of WT and G421R (*Stat3*$^{p.G421R/+}$) mice. **(B)** Stacked bar graph demonstrating the frequency of mature (TCRβ^{hi}CD24^{low}) and semi-mature (TCRβ^{hi}CD24^{+}) CD4 SP cells in the thymus (left) as well as mature (TCRβ^{+}CD24^{low}), semi-mature (TCRβ^{+}CD24^{+}), and immature (TCRβ^{-}CD24^{hi}) CD8 SP cells in the thymus (right). Bars represent mean and error bars demonstrate the SEM. **(C)** Representative flow cytometry from the thymus of 3-4 week old HOM (*Stat3*$^{p.G421R/ p.G421R}$) mice (left) and thymus cell counts (right). Cell counts from WT and G421R mice (Figure 1E) are included for comparison. **(D)** Spleen counts for young WT, G421R, and HOM mice. **(E)** Old mice pooled peripheral lymph node (axillary, brachial, inguinal) cell counts (left) and frequency of CD3^{+}CD4^{+}, CD3^{+}CD8^{+}, and CD3^{-}CD19^{+} cells within the live cell gate (right). **(F)** Representative flow cytometry from the spleen of adult mice showing Ki-67 staining in live CD3^{+}CD4^{+}CD44^{+} cells (left) and

scatter plot showing the frequency of $CD3^+CD4^+CD44^+$ Ki-67^+ cells (right). **(G)** Naïve $CD4^+$ T cells were differentiated under Th1-polarizing conditions for 5d, restimulated and stained for IFN-γ. Scatter plot showing the frequency of IFN-γ^+ cells, each dot represents the average of duplicate or triplicate wells from separate mice. **(H)** Analysis of peripheral blood reconstitution 10 weeks post-transplant, shown for WT or HOM donor mice (Ly5.2) into irradiated WT recipients (Ly5.1). Cell identification is listed on the x-axis, and frequency of Ly5.2 (donor) or Ly5.1 (recipient) cells is plotted. $n = 4$ (WT→WT) and $n = 6$ (HOM→WT). Young mice, <6wk of age; adult mice, 7-16wk of age; old mice, >20wk of age. For all scatter plots, each dot represents an individual mouse, and data are shown as mean ± SEM. Data are representative of at least 3 independent experiments, except for (F) which was 2 independent experiments. An unpaired *t* test was used for all comparisons with 2 groups, and Welch's *t* test was used in the instance of unequal variance; for those with 3 or more groups a 1-way ANOVA was used. ***$P < 0.001$, ****$P < 0.0001$.

Supplemental Figure 2

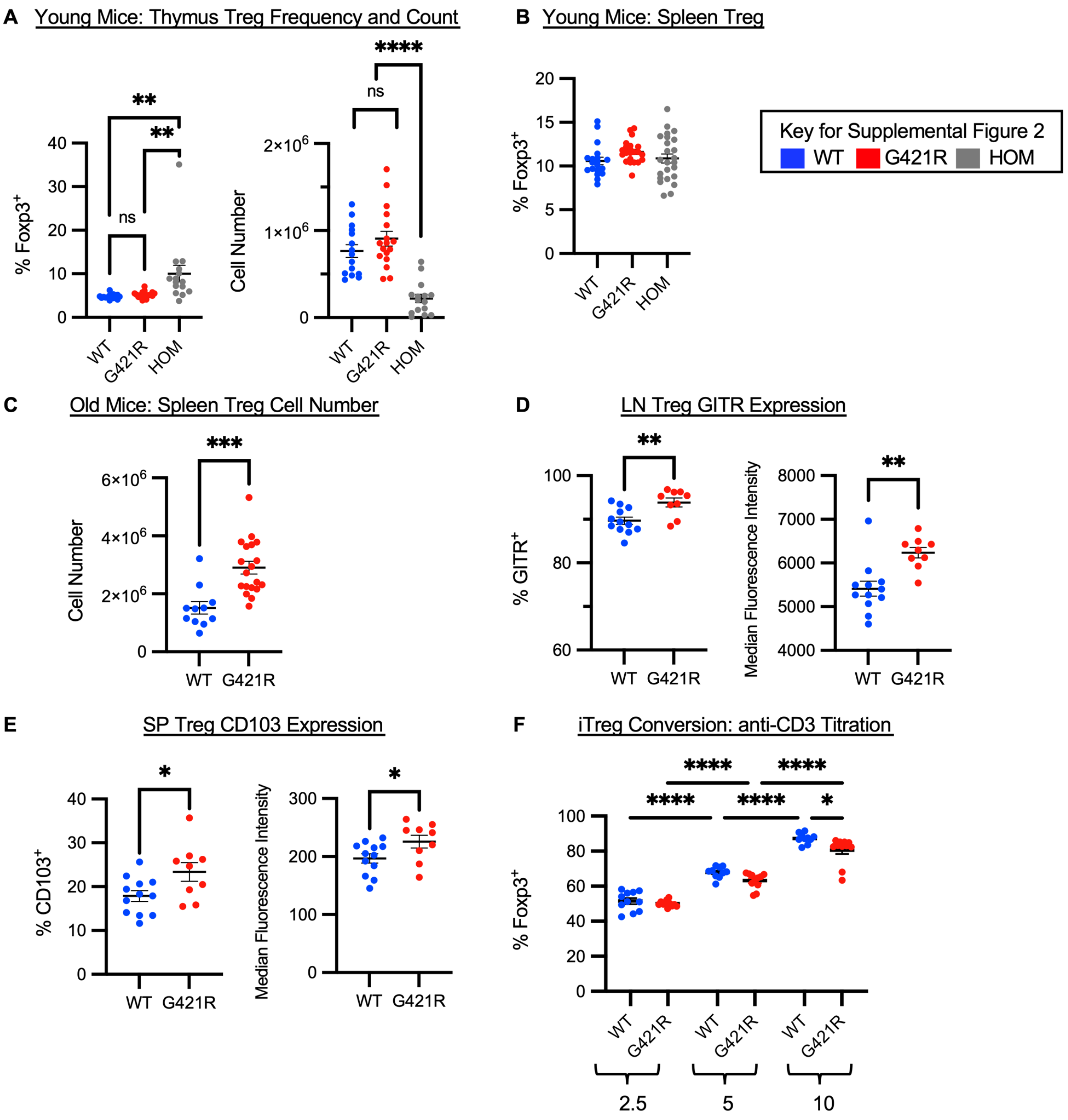

Supplemental Figure 2. Treg cell analysis in STAT3 GOF mice. (A) Frequency (left) and number (right) of $CD4^{+}CD8^{-}Foxp3^{+}$ Treg cells in the thymus of young mice. **(B)** Percentage of $CD3^{+}CD4^{+}Foxp3^{+}$ Treg cells in the spleen of young mice. **(C)** Number of $CD3^{+}CD4^{+}Foxp3^{+}$ Treg cells in the spleen of old mice. **(D)** Frequency of $GITR^{+}$ Treg cells (left) and median fluorescence intensity staining for GITR on Treg cells in the peripheral LN of WT and G421R mice. **(E)** Frequency of $CD103^{+}$ Treg cells (left) and median fluorescence intensity staining for CD103 on Treg cells in the spleen of WT and G421R mice. **(F)** Percentage of $CD4^{+}Foxp3^{+}$ Treg cells following in-vitro derived iTreg cell induction with titrating doses of anti-CD3. Young mice, <6wk of age; adult mice, 7-16wk of age; old mice, >20wk of age. For all scatter plots, each dot represents an individual mouse, and data are shown as mean ± SEM. An unpaired *t* test was used for all comparisons with 2 groups, and Welch's *t* test was used in the instance of unequal variance; for those with 3 or more groups a 1-way ANOVA was used. $*P < 0.05$, $**P < 0.01$, $***P < 0.001$, $****P < 0.0001$.

Supplemental Figure 3

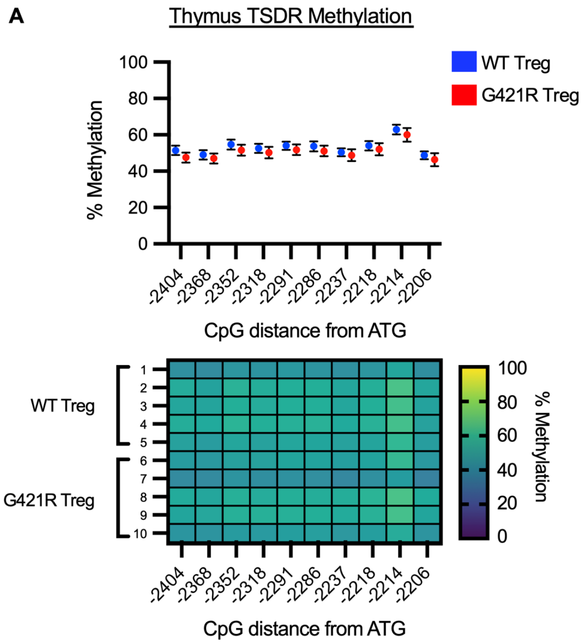

Supplemental Figure 3. STAT3 GOF Treg cells. (A) Methylation status of ten individual CpG motifs within the TSDR of CNS2 in the *Foxp3* locus. Individual CpG motifs are numbered in reference to the translational start site (ATG). The average percent methylation is shown in the dot plot (top) for WT Treg and G421R Treg isolated from the thymus of adult mice (n=5 for each group). Methylation patterns of each of the examined TSDR motifs of WT and G421R Tregs are shown in the heat map (bottom). The color code ranges from purple (no methylation) to yellow (100% methylation).

Supplemental Figure 4

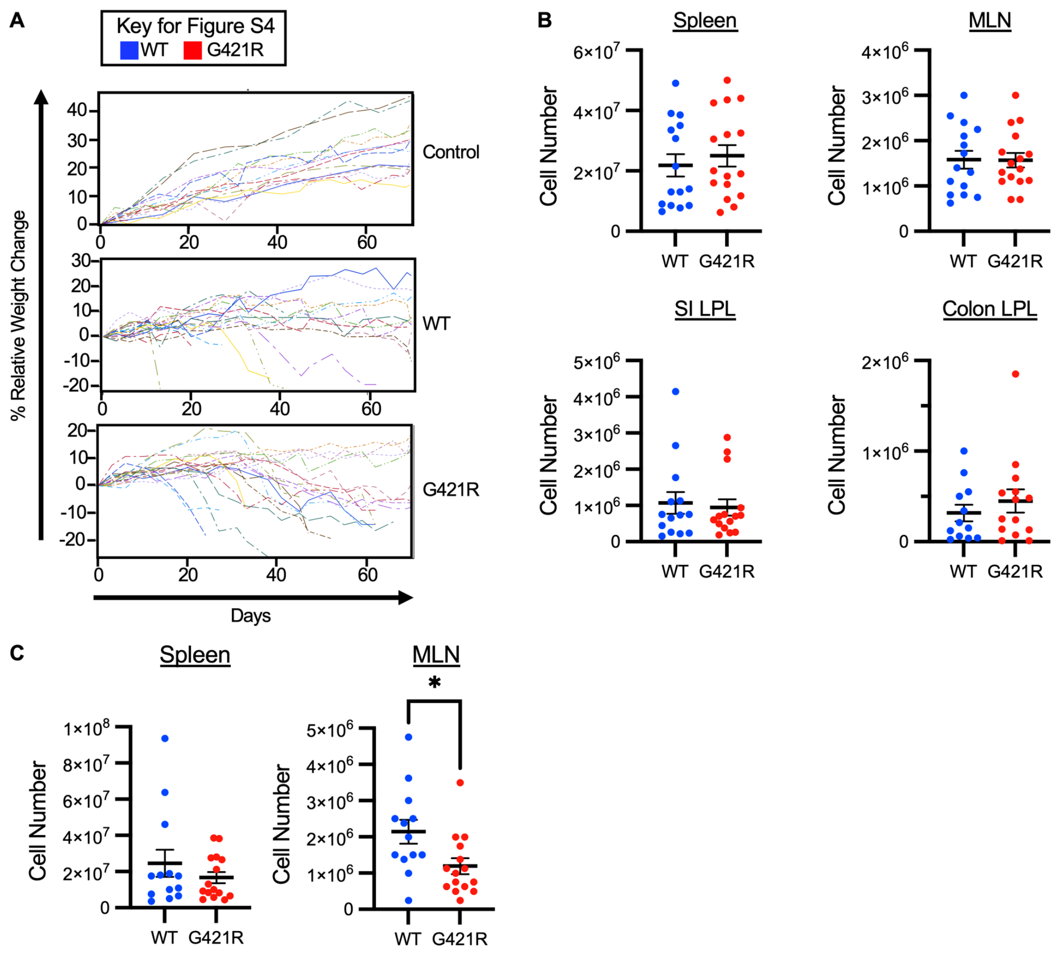

Supplemental Figure 4. STAT3 GOF T cells in a disease model. (A) Weight curves for individual mice demonstrating the percent relative weight change over time after the induction of experimental colitis with naïve T cells isolated from WT (n=20) or G421R mice (n=20) compared to control C57BL/6 *Rag1*$^{-/-}$ mice (n=14). **(B)** Cell numbers for the indicated tissues, 28 days after the induction of colitis. **(C)** Cell numbers at the conclusion of the experiment, for the indicated tissues in mice with colitis. For all scatter plots, each dot represents an individual mouse, and data are shown as mean ± SEM. An unpaired *t* test was used for all comparisons with two groups and Welch's *t* test was used in the instance of unequal variance. $*P < 0.05$.

Supplemental Figure 5

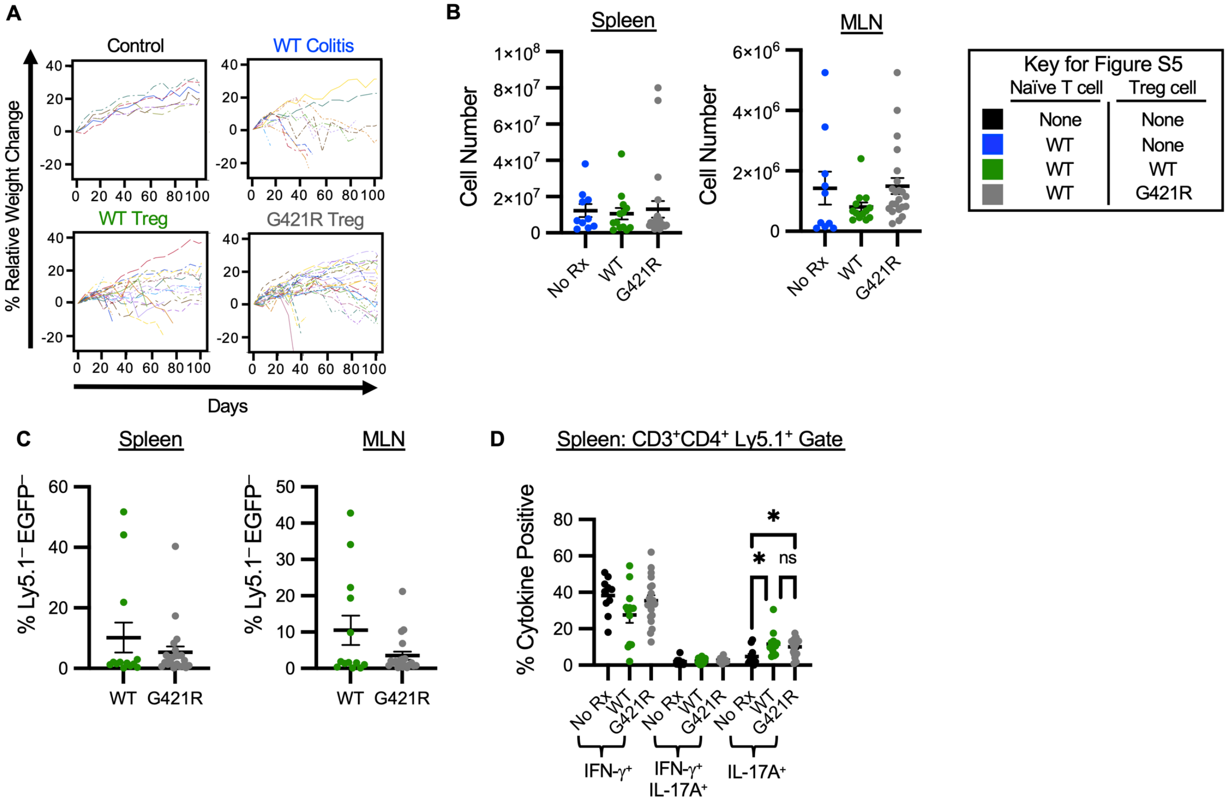

Supplemental Figure 5. STAT3 GOF Treg cells in a treatment model. (A) Weight curves for individual mice demonstrating the percent relative weight change over time after the induction of experimental colitis with naïve T cells isolated from WT mice. Mice were treated on day 21 with $1x10^6$ WT Treg cells (n=24) or G421R Treg cells (n=27) and weight patterns were compared to untreated mice (n=15) or control C57BL/6 *Rag1*$^{-/-}$ mice (n=6). **(B)** Cell numbers at the conclusion of the experiment, for the indicated tissues in mice with colitis. **(C)** Frequency of ex-Treg cells ($CD3^+CD4^+Ly5.1^-$ $EGFP^-$) in the spleen and MLN of treated mice. **(D)** Frequency of spleen $CD3^+CD4^+Ly5.1^+$ T cells that produce IFN-γ, both IFN-γ and IL-17A, or just IL-17A after stimulation with PMA/ionomycin. For all scatter plots, each dot represents an individual mouse, and data are shown as mean ± SEM. An unpaired *t* test was used for all comparisons with 2 groups, and Welch's *t* test was used in the instance of unequal variance; for those with 3 or more groups a 1-way ANOVA was used. *$P < 0.05$.

Supplemental Figure 6

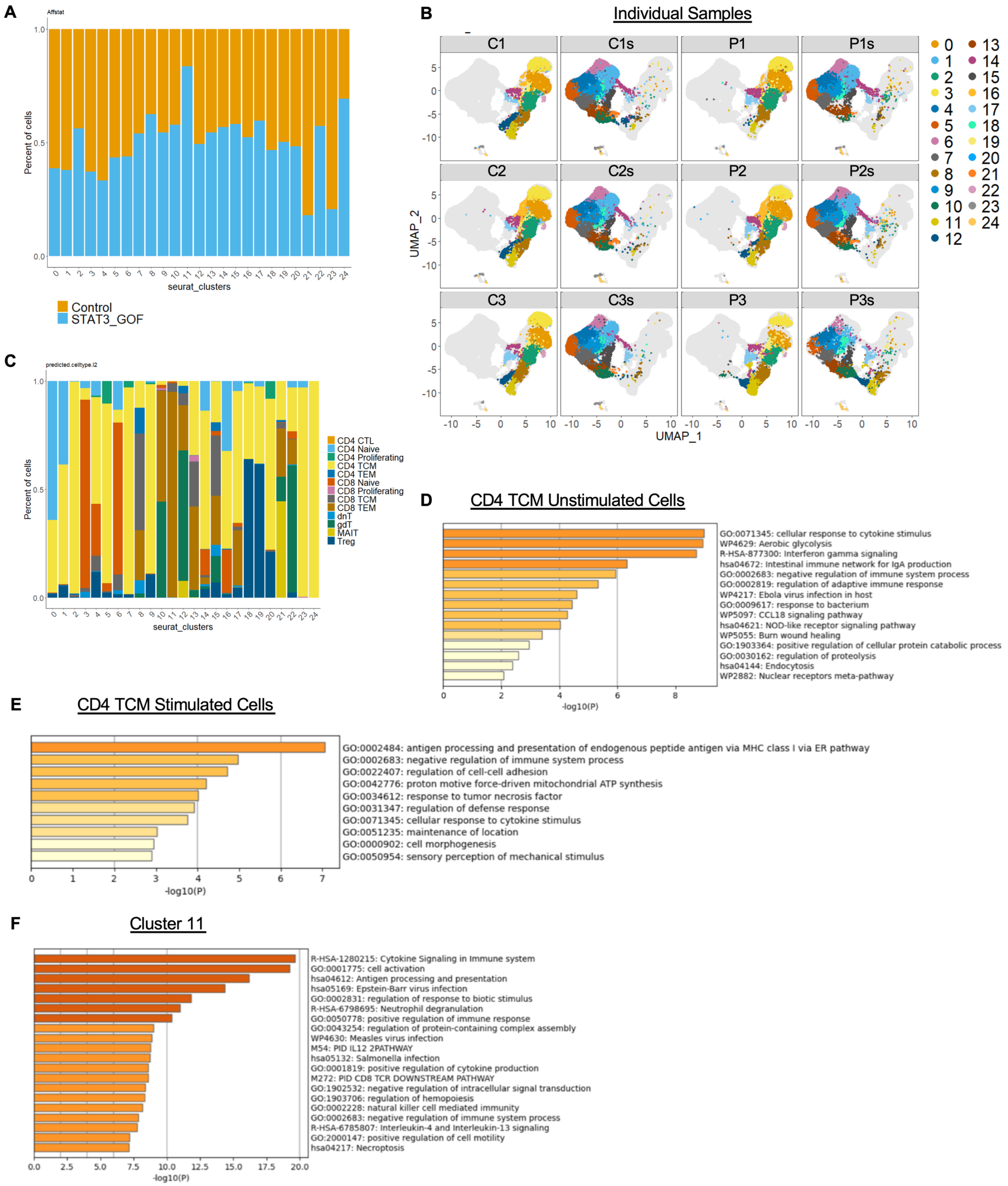

Supplemental Figure 6. Single cell RNA-sequencing of STAT3 GOF patient T cells.
(A) Cluster composition as defined by affected status (control, STAT3 GOF syndrome). **(B)** UMAP plots demonstrating Seurat clustering for each individual sample. **(C)** Stacked bar graph showing predicted cell types within each cluster. Genes that were significantly upregulated in unstimulated CD4$^+$ T central memory (TCM) cells **(D)**, stimulated CD4$^+$ TCM **(E)**, or in cluster 11 **(F)**. Differentially expressed genes (> 0.25 $\log_2$ fold change, adjusted $P < 0.05$) were analyzed for enriched terms using Metascape; top 20 most significant results shown.

Supplemental Table 1: Characteristics of STAT3 GOF Patients

	**Patient 1**	**Patient 2**	**Patient 3**
**Age, sex**	18y, male	29y, male	14y, female
**Variant**	p. T389S (c. 1165A>T)	p. R70H (c. 209G>A)	p. F174S (c.521T>C)
**Protein domain**	DNA binding domain	N-terminal domain	Coiled coil domain
**Organ involvement**	Polyarthritis, AIH, colitis, pulmonary disease, HSM, sclerotic skin, uveitis	Lymphoproliferative disease, GLILD, NRH with portal hypertension and ascites	AIH requiring liver transplant, growth failure, enteropathy, hypothyroidism, lymphoproliferation
**Immunology**	Hypogammaglobulinemia, T cell lymphopenia	Hypogammaglobulinemia, T cell lymphopenia, Evan's syndrome	Hypogammaglobulinemia, cytopenias
**Immune modulatory medications at sample**	Baricitinib, Golimumab, immunoglobulin replacement	Abatacept, immunoglobulin replacement	Tacrolimus, Prednisone, and Mycophenolate mofetil
**Other medications tried**	----	Rituximab	Rituximab, Cyclophosphamide, and Tocilizumab, Ruxolitinib (1)
**Recurrent infections**	Yes	No	Yes

AIH, autoimmune hepatitis; GLILD, granulomatous and lymphocytic interstitial lung disease; HSM, hepatosplenomegaly; NRH, nodular regenerative hyperplasia

Supplemental Methods

Generation of STAT3 GOF mice
CRISPR gRNAs for in vitro testing were identified using http://crispr.mit.edu/ and cloned into BbsI digested plasmid pX330 (addgene # 42230, gRNA sequence: 5' ATCTCTGCTCCCTAAGGGTc 3'). sgRNA activity was validated in vitro by transfection of NIH3T3 cells using ROCHE Xtremegene HP. Cell pools were harvested 48 hours later for genomic DNA prep, followed by sanger sequencing of PCR products spanning the gRNA/Cas9 cleavage site and TIDE analysis (https://tide.nki.nl/) of sequence trace files. T7 sgRNA template was PCR amplified, gel purified, and in vitro transcribed with the MEGAshortscript T7 kit (Life Technologies). T7 Cas9 template was PCR amplified, gel purified, and in vitro transcribed with the T7 mMessage mMachine Ultra kit (Life technologies). After transcription, RNA was purified with Megaclear kit (Life Technologies). A ssODN donor DNA with the mutation centered within the oligo was ordered from IDT as an ultramer oligo. Injection concentrations were: 50 ng/μl Cas9, 25 ng/μl gRNA, 20 ng/μl ssODN. ssODN sequence: 5'-gagagactggcacagcctcaagtgacacttgcctccttcttcttcctttcttccagACCCTTAGGGAGCAGAGATGTGGGAATaGAGGCCGTGCCAATTGTGATgtaagtaagttttgttcgggctaaaagcaattgttttctttctttgaggaagaaagagaaagc - 3'. Founders were identified using Qiagen Pyrosequencer and Pyromark Q96 2.5.7 software. One-cell fertilized embryos (C57/BL6 x CBA embryos) were injected into the pronucleus and cytoplasm of each zygote. Microinjections and mouse transgenesis experiments were performed as described previously (2, 3). Mice were screened using a SNP assay with primer/probe sets from ABI/Life Technologies and TaqMan Genotyping Master Mix (Thermofisher, Cat#4371355). Forward primer: CAGACCCTTAGGGAGCAGAGA, Reverse Primer: GCCCGAACAAAACTTACTTACATCAC, Reporter 1: TGTGGGAATGGAGGCC (VIC, WT sequence), Reporter 2: TGTGGGAATAGAGGCC (FAM, SNP sequence).

Phospho-flow cytometry, intracellular staining and cytokine analysis
For phospho-flow, stimulated cells were fixed with 2% paraformaldehyde and permeabilized overnight with ice-cold methanol at -20ºC. Cells were then washed 3 times with staining buffer and stained with anti-STAT3 (pY705) overnight. Intracellular cytokine staining was performed after a 4 hour restimulation with PMA (5ng/ml; Sigma-Aldrich, P1585) and ionomycin (0.5 μM; Sigma-Aldrich, I0634-1MG) in the presence of brefeldin A (1 μl/ml; BD Biosciences). Cell surface staining was performed as described and cells were fixed and permeabilized using the BD Cytofix/Cytoperm kit according to the manufacturer's instructions (BD Biosciences, Cat#554714). Cells were stained with anti-IFN-γ and anti-IL-17A antibodies for 30 minutes at 4ºC, washed and fixed with 1% paraformaldehyde for acquisition. For intranuclear staining the eBioscience Foxp3/Transcription Factor Staining Buffer set was used (Thermofisher, Cat#00-5523-00), cells were fixed and permeabilized according to the manufacturer's instructions and then stained with anti-Foxp3, anti-Ki-67, anti-Helios, or anti-CTLA4. In some experiments, Foxp3-EGFP was preserved by fixing the cells in 1% paraformaldehyde for 15 minutes prior to proceeding with the cytokine or transcription factor kit protocol.

In vitro Th1 cell differentiation assay
Sorted $CD4^+EGFP^-CD45RB^{hi}$ naïve T cells from *Foxp3*^{EGFP} and *Stat3*$^{p.G421R/+}$ *Foxp3*^{EGFP} mice ($1x10^6$/ml) were cultured in R10 media with anti-CD3 mAb (clone 145-2C11 at 3 μg/mL,BioCell Cat#BE0001-1) coated dishes in the presence of soluble anti-CD28 mAb (3 μg/mL; clone 37.51, BioCell Cat#BE0015-1), anti-IL-4 (10μg/ml; BioCell Cat#), IL-12 (10 ng/mL; PeproTech, Cat#210-12), and 5 ng/mL IL-2 (PeproTech, Cat#212-12). After 5 days, cells were restimulated with PMA and ionomycin and intracellular cytokine staining was performed as described.

Regulatory T cell suppression assay
Splenocytes from *Rag1*$^{-/-}$ mice were isolated, resuspended in R10 and plated at 50,000 cells/well in a round bottom 96-well plate. WT and STAT3 GOF Treg cells ($CD4^+EGFP^+$) cells and WT $CD4^+EGFP^-$ $CD45RB^{hi}$

naïve T cells were sorted from pooled spleens and peripheral lymph nodes as described above. WT naive T cells were labeled with TagIt violet cell tracking dye according to the manufacturer's directions. 25,000 responder CD4 T cells were plated per well with variable numbers of WT or STAT3 GOF Treg cells at a ratio of Treg:Tresp 2:1, 1:1, 1:2, 1:4, 1:8, 1:16, and 0:1. Anti-CD3 was added to a final concentration of 1 µg/mL (clone 145-2C11, BioCell Cat#BE0001-1). The cells were incubated at 37ºC for 72h and proliferation was assessed by flow cytometry. Cells were stained with anti-CD4 APC and dye dilution of TagIt violet dye and EGFP were assessed. Percent suppression was calculated by subtracting the percent proliferation of $CD4^+$ T cells for each Treg to CD4 ratio, from the percent proliferation of the $CD4^+$ T cells at the 0:1 Treg:Teff ratio, and dividing this by the percent proliferation of the $CD4^+$ T cells at the 0:1 Treg:Teff ratio.

Lamina propria digest and isolation of lymphocytes
The entire colon and the terminal 20 cm of the small intestine were isolated, flushed with PBS, cut longitudinally and washed with PBS. Sections were cut into 1 cm pieces and placed in warmed R3 media (1% Glutamine, 1% Penstrep, 3% FBS) containing DTT (Sigma, Cat#9779) and 5 mM EDTA (Fisher, Cat#BP2482100) on a shaker at 37°C for 20 min. The samples were vortexed, decanted and the incubation step was repeated. Samples were washed with RPMI plus 2 mM EDTA and then with PBS and transferred to a tube containing RPMI with DNase (Roche, Cat# 10104159001, final concentration 0.2 mg/ml) and Liberase (Roche, 05401020001, final concentration 0.0285 mg/ml). Samples were incubated for 40 min on a rocker at 37°C, cells were filtered through a 40 µm strainer and washed with R3 media containing DNase. Cells were collected by centrifugation, and then further purified by a Percoll gradient (70%/40%, Cytiva Cat#17-0891-02) and washed with PBS prior to use in downstream applications.

Bulk RNA sequencing processing and analysis
Total RNA integrity was determined using Agilent Bioanalyzer or 4200 Tapestation. Library preparation was performed with 10 ng of total RNA with a Bioanalyzer RIN score greater than 8.0. ds-cDNA was prepared using the SMARTer Ultra Low RNA kit for Illumina Sequencing (Takara-Clontech) per manufacturer's protocol. cDNA was fragmented using a Covaris E220 sonicator with peak incident power 18, duty factor 20%, cycles per burst 50 for 120 seconds. cDNA was blunt ended, had an A base added to the 3' ends, and then had Illumina sequencing adapters ligated to the ends. Ligated fragments were then amplified for 12-15 cycles using primers incorporating unique dual index tags. Fragments were sequenced on an Illumina NovaSeq-6000 using paired end reads extending 150 bases.

Basecalls and demultiplexing were performed with Illumina's bcl2fastq software and a custom python demultiplexing program with a maximum of one mismatch in the indexing read. RNA-seq reads were then aligned to the Ensembl release 76 primary assembly with STAR version 2.7.9a (4). Gene counts were derived from the number of uniquely aligned unambiguous reads by Subread:featureCount version 2.0.3 (5). Isoform expression of known Ensembl transcripts were quantified with Salmon version 1.5.2 (6). Sequencing performance was assessed for the total number of aligned reads, total number of uniquely aligned reads, and features detected. The ribosomal fraction, known junction saturation, and read distribution over known gene models were quantified with RSeQC version 4.0 (7).

All gene counts were then imported into the R/Bioconductor package EdgeR and TMM normalization size factors were calculated to adjust for samples for differences in library size (8). Ribosomal genes and genes not expressed in the smallest group size minus one samples greater than one count-per-million were excluded from further analysis. The TMM size factors and the matrix of counts were then imported into the R/Bioconductor package Limma (9). Weighted likelihoods based on the observed mean-variance relationship of every gene and sample were then calculated for all samples and the count matrix was transformed to moderated log 2 counts-per-million with Limma's voomWithQualityWeights (10). The performance of all genes was assessed with plots of the residual standard deviation of every gene to their average log-count with a robustly fitted trend line

of the residuals. Differential expression analysis was then performed to analyze for differences between conditions and the results were filtered for only those genes with Benjamini-Hochberg false-discovery rate adjusted p-values less than or equal to 0.05. Data has been deposited under accession number GSE207936.

Bisulfite conversion and methylation analysis
Bisulfite-converted DNA was prepared with the EZ DNA Methylation Kit (Zymo Research Cat# D5001). Primers were designed within the Treg cell-specific demethylated region (TSDR) chrX:7583800-7584285 (GRCm38/mm10) using MethPrimer online tool (11), forward: TGGGTTTTTTGGTATTTAAGAAAG, reverse: TTAACCAAATTTTTCTACCATTAAC, which covered 10 CpGs in this region. The region was amplified using the Qiagen Pyromark PCR Kit according to the manufacturer's directions (Qiagen Cat#978703). Band size was verified by diagnostic gel (amplicon coordinates ChrX:7583918-7584173, 256bp). PCR products were purified with Monarch PCR DNA Cleanup Kit (Cat#T1030S). Illumina Y-adapters were ligated onto the PCR products using NEB Quick ligase. Ligation was verified by diagnostic gel and the ligation products were purified with Monarch DNA Gel Extraction Kit (NEB). Index primers were added while amplifying the adapter-ligated products using NEB Phusion High Fidelity DNA Polymerase and Illumina primer 1.0, and primer 2.0, and a standard index primer with custom indices. The indexed PCR product was verified by diagnostic gel and purified using Monarch DNA Gel Extraction Kit (NEB). DNA libraries were submitted for paired-end 250 bp read next generation sequencing. FASTQ data files were trimmed using trimGalore (https://www.bioinformatics.babraham.ac.uk/projects/trim_galore/) with the –trim-n, --paired, and –q 30 flags. Reads were mapped to the in silico converted reference sequence using Bismark and the –bowtie2 flag to obtain the CpG coordinates, percent methylation, and the coverage (12).

Supplemental Table 2: Summary of Antibodies and Dyes

Target	Clone	Fluorophore	Manufacturer	Cat#
B220	RA3-6B2	BUV395	BD Biosciences	563793
B220	RA3-6B2	BV510	Biolegend	103247
CD3ε	145-2C11	PerCP-Cy5.5	BD Biosciences	551163
CD3ε	145-2C11	APC-Cy7	Biolegend	100330
CD3ε	145-2C11	BV785	Biolegend	100355
CD4	RM4-5	Pacific Blue	BD Biosciences	558107
CD4	GK1.5	BV510	Biolegend	100449
CD4	GK1.5	APC	Biolegend	100412
CD4	GK1.5	BUV395	BD Biosciences	563790
CD4	RM4-5	Alexa Fluor 700	BD Biosciences	557956
CD4	RM4-5	APC	BD Biosciences	553051
CD8α	53-6.7	BV510	BD Biosciences	563068
CD8α	53-6.7	APC	BD Biosciences	553035
CD8α	53-6.7	BUV563	BD Biosciences	748535
CD11b	M1/70	Alexa Fluor 700	Biolegend	101222
CD11b	M1-70	FITC	Biolegend	101206
CD11c	HL3	APC-Cy7	BD Biosciences	561241
CD19	1D3	BUV395	BD Biosciences	563557
CD19	6D5	PE-Dazzle 594	Biolegend	115554
CD24	M1/69	PE/Cyanine7	Biolegend	101822
CD25	PC61	BV786	BD Biosciences	564023
CD25	PC61	PE	Biolegend	102007
CD25	PC61	APC	BD Biosciences	557192
CD44	IM7	APC	Biolegend	103012
CD44	IM7	PE	Biolegend	103007
CD45.1	A20	BV421	Biolegend	110732
CD45.1	A20	APC	BD Biosciences	558701
CD45.1	A20	BUV395	BD Biosciences	565212
CD45.2	104	PerCP-Cy5.5	BD Biosciences	552950
CD45RB	16A	Alexa Fluor 647	BD Biosciences	562848
CD62L	MEL-14	PE	Biolegend	104408
CD62L	MEL-14	FITC	Biolegend	104406
CD103	2E7	Pacific Blue	Biolegend	121418
CTLA-4	UC10-4B9	APC	Biolegend	106309
F4/80	BM8	PE	Biolegend	123109
Foxp3	FJK-16s	PE	Invitrogen	12-5773-82
GITR	DTA-1	APC	Invitrogen	17-5874-81
Helios	22F6	Pacific Blue	Biolegend	137210
IFN-γ	XMG1.2	PE	Biolegend	505808
IL-17A	TC11-18H10	Alexa Fluor 647	BD Biosciences	560184
Ki-67	B56	Alexa Fluor 647	BD Biosciences	561126
KLRG1	2F1	PE	BD Biosciences	561621
Ly6C	HK1.4	Pacific Blue	Biolegend	128014
Ly6G	1A8	BV711	Biolegend	127643
NK1.1	PK136	PE/Cyanine7	Biolegend	108714

STAT3 (pY705)	4/P-STAT3	Alexa Fluor 647	BD Biosciences	557815
TCRb	H57-597	Alexa Fluor 700	Biolegend	109224
TCRγδ	GL3	BV650	BD Biosciences	563993
Zombie Yellow Dye			Biolegend	77168
TagIt Violet			Biolegend	425101

References

1. Forbes LR, et al. Jakinibs for the treatment of immune dysregulation in patients with gain-of-function signal transducer and activator of transcription 1 (STAT1) or STAT3 mutations. *J Allergy Clin Immunol.* 2018;142(5):1665-9.
2. *Advanced Protocols for Animal Transgenesis.* Berlin, Heidelberg: Springer Berlin, Heidelberg; 2011.
3. Behringer R, et al. *Manipulating the mouse embryo: a laboratory manual.* Cold Spring Harbor, New York: Cold Spring Harbor Laboratory Press; 2014.
4. Dobin A, et al. STAR: ultrafast universal RNA-seq aligner. *Bioinformatics.* 2013;29(1):15-21.
5. Liao Y, et al. featureCounts: an efficient general purpose program for assigning sequence reads to genomic features. *Bioinformatics.* 2014;30(7):923-30.
6. Patro R, et al. Salmon provides fast and bias-aware quantification of transcript expression. *Nat Methods.* 2017;14(4):417-9.
7. Wang L, et al. RSeQC: quality control of RNA-seq experiments. *Bioinformatics.* 2012;28(16):2184-5.
8. Robinson MD, et al. edgeR: a Bioconductor package for differential expression analysis of digital gene expression data. *Bioinformatics.* 2010;26(1):139-40.
9. Ritchie ME, et al. limma powers differential expression analyses for RNA-sequencing and microarray studies. *Nucleic Acids Res.* 2015;43(7):e47.
10. Liu R, et al. Why weight? Modelling sample and observational level variability improves power in RNA-seq analyses. *Nucleic Acids Res.* 2015;43(15):e97.
11. Li LC, and Dahiya R. MethPrimer: designing primers for methylation PCRs. *Bioinformatics.* 2002;18(11):1427-31.
12. Krueger F, and Andrews SR. Bismark: a flexible aligner and methylation caller for Bisulfite-Seq applications. *Bioinformatics.* 2011;27(11):1571-2.
